# Eye-tracking as a proxy for coherence and complexity of texts

**DOI:** 10.1371/journal.pone.0260236

**Published:** 2021-12-13

**Authors:** Débora Torres, Wagner R. Sena, Humberto A. Carmona, André A. Moreira, Hernán A. Makse, José S. Andrade

**Affiliations:** 1 Departamento de Física, Universidade Federal do Ceará, Fortaleza, Ceará, Brazil; 2 Levich Institute and Physics Department, The City College of New York, New York City, New York, United States of America; Universidade Estadual de Maringa, BRAZIL

## Abstract

Reading is a complex cognitive process that involves primary oculomotor function and high-level activities like attention focus and language processing. When we read, our eyes move by primary physiological functions while responding to language-processing demands. In fact, the eyes perform discontinuous twofold movements, namely, successive long jumps (saccades) interposed by small steps (fixations) in which the gaze “scans” confined locations. It is only through the fixations that information is effectively captured for brain processing. Since individuals can express similar as well as entirely different opinions about a given text, it is therefore expected that the form, content and style of a text could induce different eye-movement patterns among people. A question that naturally arises is whether these individuals’ behaviours are correlated, so that eye-tracking while reading can be used as a proxy for text subjective properties. Here we perform a set of eye-tracking experiments with a group of individuals reading different types of texts, including children stories, random word generated texts and excerpts from literature work. In parallel, an extensive Internet survey was conducted for categorizing these texts in terms of their complexity and coherence, considering a large number of individuals selected according to different ages, gender and levels of education. The computational analysis of the fixation maps obtained from the gaze trajectories of the subjects for a given text reveals that the average “magnetization” of the fixation configurations correlates strongly with their complexity observed in the survey. Moreover, we perform a thermodynamic analysis using the Maximum-Entropy Model and find that coherent texts were closer to their corresponding “critical points” than non-coherent ones, as computed from the Pairwise Maximum-Entropy method, suggesting that different texts may induce distinct cohesive reading activities.

## Introduction

Understanding how people capture and assimilate written information involves multiple fields of science, from human anatomy and neurology to linguistics. In particular, the eye movement has always been of interest for the comprehension of reading behavior, since it represents an observable link between the mechanics of vision and its cognitive activity.

In the 19th century, the founders of visual-behavior research examined eye movement in elementary experiments and described how the eyes capture visual information for processing in the brain [1]. In these experiments, it was noticed that the eyes do not register information through smooth continuous movements; instead, they make successive jerks (saccades), between events in which the gaze is briefly maintained on small confined regions (fixations) [2–6]. Moreover, we move our eyes in such a way that, through fixations of precise duration and location, they can efficiently capture pieces of visual data that our brain then puts together in order to create a complete neat image. In this way, the mental demands in processing the image also influences where we sequentially direct the gaze. With the development of eye-tracking devices, scientists were able to observe gaze trajectories and it became clear that the eye movement also depends on the attention focus and examination strategies [6–9].

In the late 20th century, a new area of research in linguistics focused on studying eye movements while reading. In these studies, different strategies were applied in order to analyze how words are fixated depending on specific linguistic factors. It was found that both the number and duration of the fixations on each word are plausible measures to quantify word processing and language comprehension [10–13]. It has been generally accepted that the properties of a given word which are significantly correlated with read processing are its frequency in the language [11, 12, 14–17], length [12, 15, 18] and predictability in context [12, 15, 17, 19, 20]. Under the same framework, eye-tracking experiments have been successfully combined with mathematical models to formally describe how individuals control eye movement while reading, with attention shifting from word to word [21–24].

Complexity and coherence of a text are considered main linguistic attributes to evaluate reading comprehension and learning difficulties [25, 26]. Linguistic researchers have been focusing on measuring texts complexity over the last decades [27]. Mainly, the issue has gained importance due to the need to select appropriate texts for different scholarly levels that would allow students to progressively develop reading and text comprehension skills [25]. For this reason, mathematical expressions for readability and metrics have been developed in order to quantify the complexity of texts and categorize reading material [28]. The variables frequently used are the average length of the words and their frequency in the language, both accounting for semantic difficulty, as well as sentence length, which is closely related to syntactic complexity. The relevant premise behind these empirical expressions is rather obvious, namely, that texts with unusual, long words and extensive sentences are more difficult to process than texts with familiar vocabulary and short sentences. In contrast, the coherence of a text is related to its meaningfulness, a notion associated with semantics rather than grammatical structure [29]. A coherent text makes sense in such a way that the ideas in it are continually connected and the text is consistent as a whole. Its sentences not only have meaning on their own, but, more importantly, they successively build on the meaning of the text.

It is expected that features of a text such as genre and style may be reflected in the eye movement patterns of individuals when reading text passages. Different types of texts may therefore prompt different reading responses in terms of fixation configurations and, consequently, different cognitive reactions. Thus, in order to study this interplay, a phenomenological modeling approach based on eye-movement data (*i.e*., fixation patterns) should be able to capture the inner cognitive processes underlying reading. In this regard, models from Statistical Physics such us the Maximum-Entropy Model (MEM) developed in information theory can provide a statistical conceptual framework to understand a given natural process in terms of the “interactions” among its many elementary units using statistical data obtained experimentally [30–32]. The principle of maximum entropy states that the probability distribution that best represents the state of a given system is the one that maximizes its entropy, being also in conformity with one or a set of specific constraints. This principle, by itself, contains the essence of the so called *Inverse Ising Problem* solution, in which the Hamiltonian (*i.e*., the interactions) in a given complex system can be inferred from observed statistical correlations among its components. This statistical analysis is frequently referred to as the *Boltzmann-machine*, since it uses the Boltzmann distribution in its core.

The MEM approach has been applied to a wide variety of systems which can be mapped to Ising-like models. In this representation, the interacting elements can be in an active or inactive state, analogously to an Ising type system (*i.e*., a lattice of dipole moments in which the spins are in either up, +1, or down, −1, states that can be under the action of an external field). In the case of neuronal networks, for example, the interactions between neurons subjected to some stimuli are inferred from the collected data of their firing patterns [33–36]. In a larger scale, the interactivity among regions of the human brain, for example, has been investigated from data of nuclear magnetic resonance [37]. An important application of the MEM is the characterization of protein-protein interaction benefiting from large protein databases [38, 39]. In particular, this strategy has been used to infer genetic interaction networks from known gene expression patterns [40–42]. The collective response exhibited by flocks of birds was also studied by means of the MEM [43, 44] as well as the emergence of collective behavior from the eye movement patterns of a group of people while watching commercial videos [45]. In the last case, pairwise correlations among series of instantaneous eye’s velocities were utilized to capture the collective response of the individuals and relate it to video popularity. Finally, the MEM approach has also proved to be effective in other fields outside biology. In [46], for example, the intricate network micro-structure of interactions in the stock market has been captured through pairwise correlations calculated from big data bases of stocks variability.

**Fig 1:**
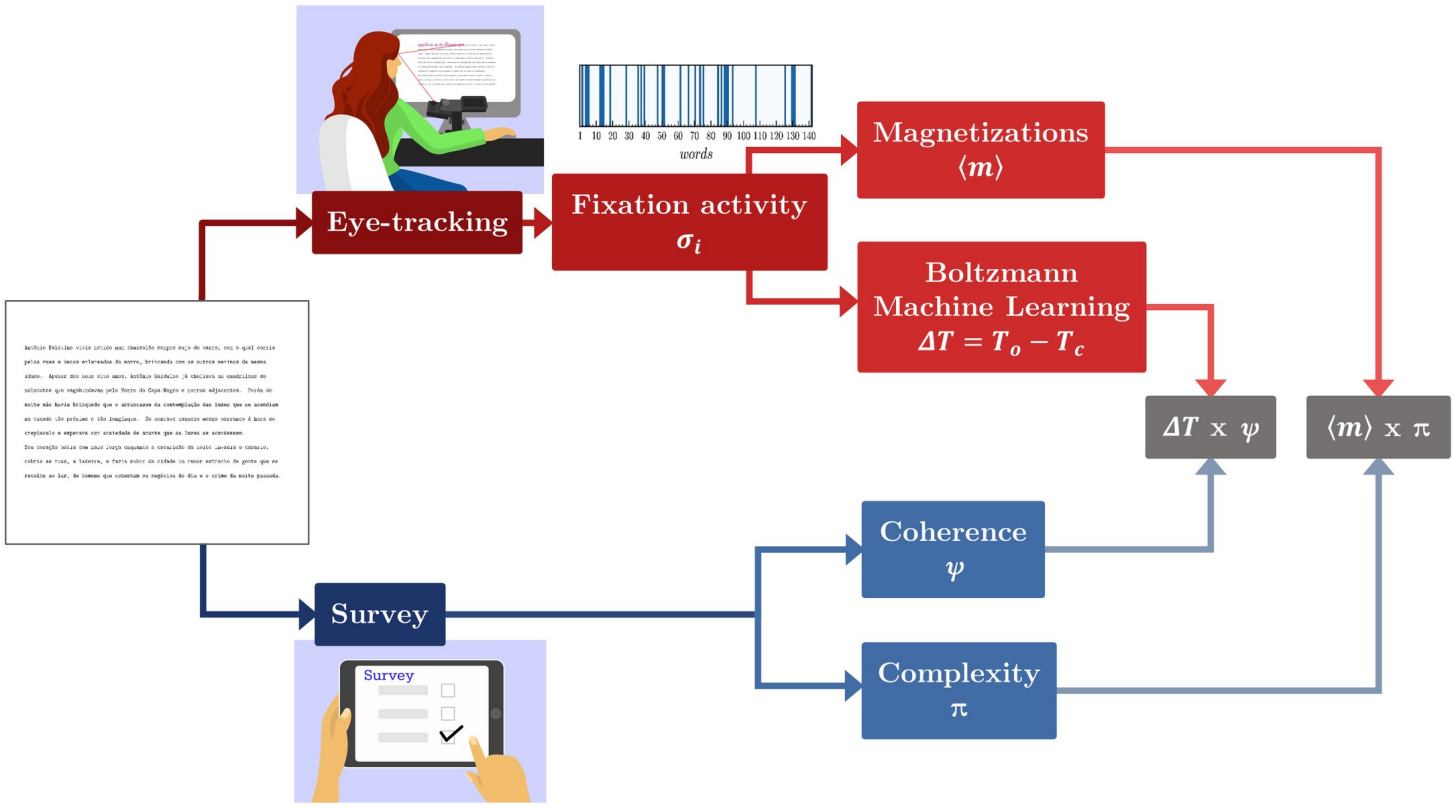
Diagram of the research route. We take a twofold approach to characterize through the cognitive reading activity the complexity and coherence of texts. On one side, we perform an eye-tracking experiment to collect fixation data from a group of people. The fixation activity associated to each subject while reading a given text is computed by binarizing the states of each word, defined positive +1, if the subject fixates it at least twice, or negative −1 if the subject does not fixate or fixates it only once. We then compute the reading “magnetization” of a given text for each subject and average it over all subjects to obtain 〈*m*〉. From the pairwise cross-correlations between the fixation sequences of the subjects, we also infer a “Hamiltonian” for each text by means of the Maximum Entropy principle using a Boltzmann machine-learning algorithm. A thermodynamic analysis of the energy fluctuations allows us to determine whether the text is near a “critical point”. In parallel, we collected reading-comprehension data from an Internet extensive survey performed with 400 people in an attempt to quantify the complexity 〈*π*〉 and coherence 〈*ψ*〉 of the texts.

As shown in Fig 1, here we address the problem of characterizing the complexity and coherence of diverse texts quantitatively by taking a twofold approach. First, we perform eye-tracking experiments with a limited group of people to directly analyze their fixation data while reading different texts, namely, children stories, excerpts from literary works and random word generated texts (see Table 1). This is achieved by expressing the experimental results in terms of a binary model for fixation sequences (analogous to an Ising system) which is duly embedded in the MEM. It enables us to disclose two indexes that can be used as potential proxies for complexity and coherence: the magnetization (a measure of the density of the fixation sequences) and the distance between the “operating temperature” of the system and its critical temperature (a measure of cohesion among the fixation sequences). Second, our experimental approach is then validated through an extensive Internet reading survey, with access to a vast respondent sample, to categorize the same texts according to different complexity and coherence levels, therefore permitting a direct comparison with the obtained eye-tracking indexes.

**Table 1:** Texts information.

Symbol	Title	Author	Year	Country
GAU	O Gaúcho	José de Alencar	1870	Brazil
GSV	Grande Sertão: Veredas	João Guimarães Rosa	1956	Brazil
HCL	História do Cerco de Lisboa	José Saramago	1989	Portugal
JUB	Jubiabá	Jorge Amado	1935	Brazil
MEL	A Mão e a Luva	Machado de Assis	1874	Brazil
QUI	O Quinze	Rachel de Queiroz	1930	Brazil
RT1	Random text 1	-	-	-
RT2	Random text 2	-	-	-
ST1	Story 1: A patinha Esmeralda	-	-	Brazil
ST2	Story 2: A menina do leite	-	-	Brazil

Basic information about the 10 texts used in the eye-tracking experiments. All texts are written in Portuguese. RT1 and RT2 texts are generated with an online random word generator [47]. ST1 and ST2 texts are popular children stories of unknown author and year.

## Materials and methods

### Eye-tracking reading experiment

#### Methodology

The experiments were conducted using a SR Research EyeLink 1000 eye tracker, with the Desktop Mount Participant Setup. It operates at a sampling frequency of 1 kHz using a monocular device and an infrared video-based eye tracker [48]. This equipment is based on the Pupil Center Corneal Reflection system (PCCR) [49, 50], one of the most accurate, non-intrusive eye-tracking techniques. When a stimuli is presented to the subject on a display monitor, near infrared light is shined onto the subjects’ eyes and the reflections are recorded with a special camera. Part of the light is reflected in the cornea, appearing as a small, sharp glint (known as the “first Purkinje image”), and another part reaches the retina and reflects back making the pupil appear as a bright, well defined disc (“bright pupil” effect). The reflected images are captured by the camera and are then processed by the EyeLink software. The vector between the pupil and corneal reflections is used to calculate the exact gaze location of each sample.

We selected 20 participants among physics and engineering graduate and postgraduate students with ages from 17 to 34, all Brazilian Portuguese native speakers. The reading material consisted of 10 different types of texts (all in Portuguese), including two children stories, two random word generated texts (with standard grammatical structure, but random content) [47] and six excerpts from literature work (see Table 1). All texts were in 12 point size mono-spaced font. For our equipment setup, these characteristics are compatible with the condition that a visual angle of 1° spans a length of 3 characters, which gives word position accuracy [50]. Letters were light cyan and the background dark gray, which provides high color contrast and moderate brightness in order to ensure readability while improving the eye-tracking accuracy.

The eye-tracking calibration process consists in collecting raw eye data when the subject fixates at target points, presented one by one at the display monitor. Next, the information is processed and the gaze positions are calculated. The offset between the sampled gaze and the displayed point positions determines the quality of calibration. This protocol was followed before each reading for every participant. A validation test was then performed after calibration to confirm that its accuracy was always within the error range from 0.25° to 1°.

**Fig 2:**
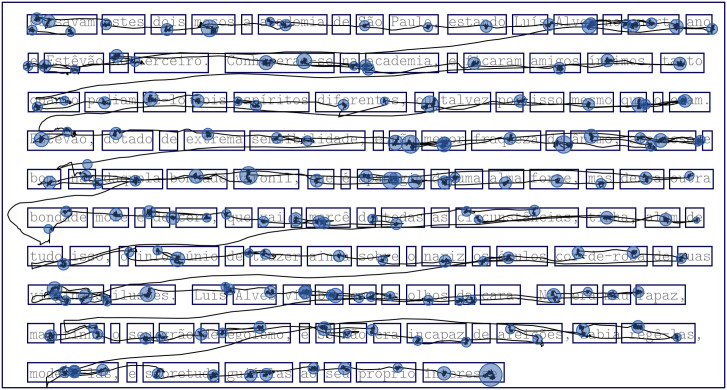
Eye-tracking reading pattern. Plot showing the sequences of gazes and fixations during a typical eye-tracking experiment. In this particular case, the data was collected while the subject was reading the MEL text. The blue circles represent the fixations and their sizes stand for the corresponding duration times. The solid lines between circles indicate the gaze trajectory along the text. For a given text, we measure the number of times $n_{i}^{r}$ during the entire reading that a fixation of subject *i* falls into the rectangle box delimiting a word *r*.

Before starting the experiment, with the purpose of motivating the participants to read consciously, they were warned that we would ask them to answer a simple question after reading each text. To run the experiment, the subject seated in front of a display screen and the head was stabilized by the use of an adjustable head and chin rest. Without imposing any time limit for reading, the texts were sequentially shown on the screen, intercalated by their corresponding questions. During the reading session, the eye tracker collected gaze location data at a sample rate of 1000 Hz, which gives an average temporal error of 0.5 ms (approximately half the duration of the time between samples) [50]. The collected data was de-identified in order to preserve the participants’ privacy. The experiment was designed using the SR Research Experiment Builder program (version 1.10.1630) and the collected data was displayed and filtered with the Eye-Link Data Viewer software (version 1.10.1). The reading data is processed by first delimiting each word in the texts with rectangles. For each subject reading a given text, we obtain the spatial coordinates of the fixations from the eye tracker and count how many of them fall into each box, as exemplified in Fig 2. At the end of the experiment, an array can be associated to each text, whose elements $n_{i}^{r}$ correspond to the number of fixations that a given word *r* received from the subject *i*. In addition, the time duration of each reading was simultaneously registered during every eye-tracking experiment.

#### Ethics

The eye-tracking experiment procedures were approved by the Research Ethics Committee of the Federal University of Ceará (COMEPE, Universidade Federal do Ceará, Brasil). All subjects gave written informed consent. Also, parental consent was obtained from the parents of the minors included in the study.

#### Fixation activity model

In order to make possible the analogy with the Ising system, we define the fixation activity $\sigma_{i}=\left\{\sigma_{i}^{1},\ldots ,\sigma_{i}^{M}\right\}$ for each subject *i* reading a given text with *M* words in terms of the state of each word *r* $\sigma_{i}^{r}=\pm 1$ according to the following rule,

$$\begin{array}{c} \sigma_{i}^{r}=\{\begin{array}{cc} +1 & \text{if}\;n_{i}^{r}\geq 2\\ -1 & \text{if}\;n_{i}^{r}<2\;, \end{array} \end{array}$$ (1)

where $n_{i}^{r}$ represents the number of times the subject *i* fixates on word *r* during the reading. The value of 2 fixations per word has been adopted here as threshold parameter to define whether a word is active or not in the text due to the fact that, from our eye-tracking experiments, almost every word in any text was fixated at least once during the readings of all subjects. This reading pattern is compatible with observations reported previously [10]. Thus, relevant variations among the fixation activities would be detected considering the words with one fixation and those with two or more.

**Fig 3:**
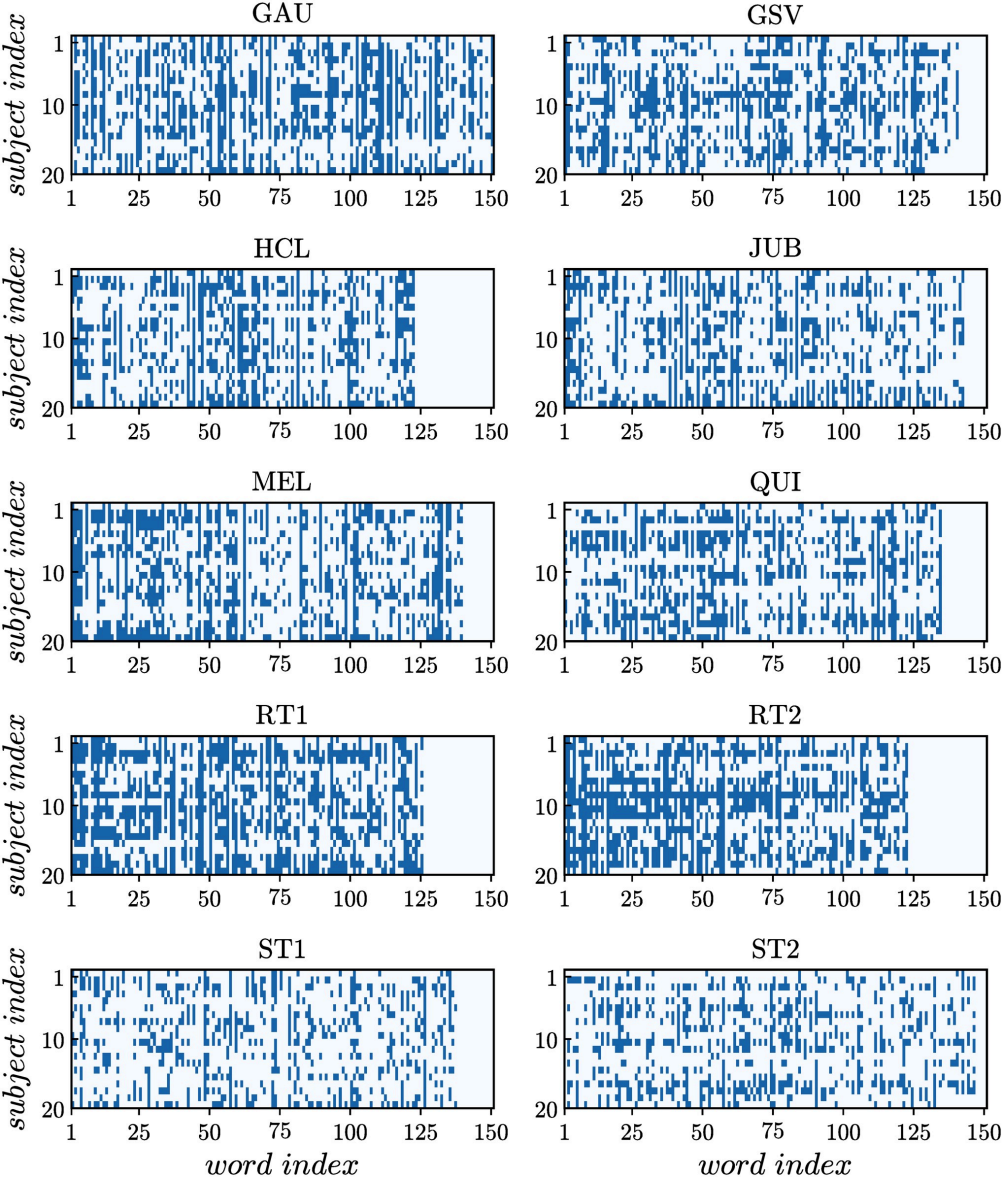
Fixation activities. Raster plots of the fixation activities obtained for all subjects while reading the texts. Accordingly, for each subject *i*, the state $\sigma_{i}^{r}$ of a word is active (+1) if $n_{i}^{r}\geq 2$ (blue) or inactive (−1) if $n_{i}^{r}<2$ (white).

The raster plots corresponding to the fixation activities obtained from the eye-tracking experiments with all subjects are shown in Fig 3 for all the texts. Clear differences in the reading activity patterns can be observed. In particular, we notice that the density of active states observed for ST1 and ST2 is significantly lower than for RT1 and RT2. The fixation activity density is quantified here in terms of the “magnetization” *m*_*i*_ for each subject *i* = 1, …, *N*, defined as the average of the fixation states $\sigma_{i}^{r}$ over the *M* words of the text,

$$\begin{array}{c} m_{i}=\left\langle \sigma_{i}\right\rangle =\frac{1}{M}\sum_{r=1}^{M}\sigma_{i}^{r}\;. \end{array}$$ (2)

In this way, for every text, we can define an overall magnetization as,

$$\begin{array}{c} \left\langle m\right\rangle =\frac{1}{N}\sum_{i=1}^{N}m_{i}\;. \end{array}$$ (3)

A relevant measure that could be readily obtained from the experiments performed here, being certainly indicative of text processing demand, is the average reading time per word of the text,

$$\begin{array}{c} \left\langle t\right\rangle =\frac{1}{N}\sum_{i=1}^{N}t_{i}/M\;, \end{array}$$ (4)

where *t*_*i*_ is the reading time of subject *i*. The values of the average magnetization 〈*m*〉 and average reading time per word 〈*t*〉 obtained from the experiments for every text are reported in Table 2. Accordingly, the reading of ST1 and ST2 texts resulted in the two lowest values of both measures. Also, the similar values obtained for RT1 and RT2 that are, however, somewhat higher than for most of the other texts demonstrates some degree of correlation between the two measures. Despite the similarities, 〈*m*〉 and 〈*t*〉 are not perfectly compatible. As a matter of fact, in what follows we will show that 〈*m*〉 captures information on the cognitive activity while reading more subtly than 〈*t*〉 does.

**Table 2:** Average magnetizations and reading times per word.

Text	〈*m*〉	〈*t*〉 (ms)
GAU	-0.2147	744.82
GSV	-0.2657	611.12
HCL	-0.2918	595.08
JUB	-0.3697	552.34
MEL	-0.2460	641.70
QUI	-0.3022	513.37
RT1	-0.0464	923.98
RT2	-0.0664	793.13
ST1	-0.5190	398.38
ST2	-0.4966	394.21

The magnetization 〈*m*〉 and reading time per word 〈*t*〉 of each text correspond to the average values of the reading fixation activity and time per word, respectively, also averaged over all subjects.

### Maximum Entropy Model

We model the data obtained from our eye-tracking experiments following the Maximum Entropy principle [31] considering a system of binary variables (the fixation activities) with pairwise couplings [32]. Let us denote $\sigma =\{\sigma_{1}^{r},\ldots ,\sigma_{N}^{r}\}$ the state of the system consisting of *N* subjects reading word *r* in a given text. Since every subject can only be in one of two states (+1 or −1), overall we have a set {*σ*} of 2^*N*^ possible states that the system can occupy, for each word in the text. Next, we calculate the covariance *C*_*ij*_ between the fixation activities, for every pair of subjects *i* and *j* along the *M* words of the text,

$$\begin{array}{c} C_{ij}=\left\langle \sigma_{i}\sigma_{j}\right\rangle -\left\langle \sigma_{i}\right\rangle \left\langle \sigma_{j}\right\rangle \;, \end{array}$$ (5)

where

$$\begin{array}{c} \left\langle \sigma_{i}\sigma_{j}\right\rangle =\frac{1}{M}\sum_{r=1}^{M}\sigma_{i}^{r}\sigma_{j}^{r}\;, \end{array}$$ (6)

and 〈*σ*_*i*_〉 is given by Eq (2). The minimal probability distribution *P*({*σ*}) that represents our system is the one that maximizes the entropy while reproducing our observations, *i.e*., the average *m*_*i*_ and covariance *C*_*ij*_ for all *i* and *j*. Subject to these constraints, the form of *P* is the Boltzmann’s probability distribution [31] (see S1 Appendix),

$$\begin{array}{c} P\left(\left\{\sigma \right\}\right)∼{\text{e}}^{-E/T}, \end{array}$$ (7)

where *T* is analogous to a temperature and *E* to a Hamiltonian. This distribution results as the least biased representation for an Ising-type system like ours, with known first and second moments. Specifically, as a first approximation, the energy term has the same form of the Ising model [32],

$$\begin{array}{c} E=-\sum_{i=1}^{N}h_{i}\sigma_{i}-\sum_{i,j=1}^{N}J_{ij}\sigma_{i}\sigma_{j}. \end{array}$$ (8)

This mathematical correspondence naturally lead us to interpret *h*_*i*_ as the action of a local external stimulus (text) on subject *i*, analogous to a “random field”, and *J*_*ij*_ as “coupling coefficients” between subjects *i* and *j*. Although the participants never really communicate with each other in our eye-tracking experiments, we can think of the text as a medium through which subjects *i* and *j* “interact”. This means that, although the subjects read the texts individually, their fixation activities may relate to each other given similarities in their cognitive responses induced by the characteristics of the texts. These pairwise couplings or interactions between the subjects reading activities give rise to the observed correlations among them. Consequently, the correlations may lead to emergent collective effects, which can be of importance for the study of the system.

At this point, we seek compute the local fields *h*_*i*_ and the interactions *J*_*ij*_ by directly solving the inverse problem given by Eq (8) (see S1 Appendix). Once we infer the values of *h*_*i*_ and *J*_*ij*_ for all subjects that better reproduce the experimentally observed magnetizations *m*_*i*_ and covariances *C*_*ij*_, while maximizing the entropy, the Boltzmann probability distribution of Eq (7) characterizes the statistics of each text. For simplicity, here we arbitrarily set the “operating temperature”, namely, the reading temperature, *T*_*o*_ = 1. By doing so, from Eq (7) it is possible to compute the rate in which the average energy of a given text changes with *T*,

$$\begin{array}{c} C_{v}=\frac{\partial {\left\langle E\right\rangle }_{P}}{\partial T} \end{array}$$ (9)

This rate of change is analogous to a heat capacity, *i.e*., a measure of how much energy the system can absorb as the temperature *T* increases. Moreover, at a “critical temperature” *T*_*c*_, *C*_*v*_ is maximal, which is interpreted as a phase transition: if *T*_*c*_ < *T*_*o*_, the system is in a “liquid” or random state. On the other hand, *T*_*c*_ > *T*_*o*_ is indicative of a more “ordered” condition.

**Fig 4:**
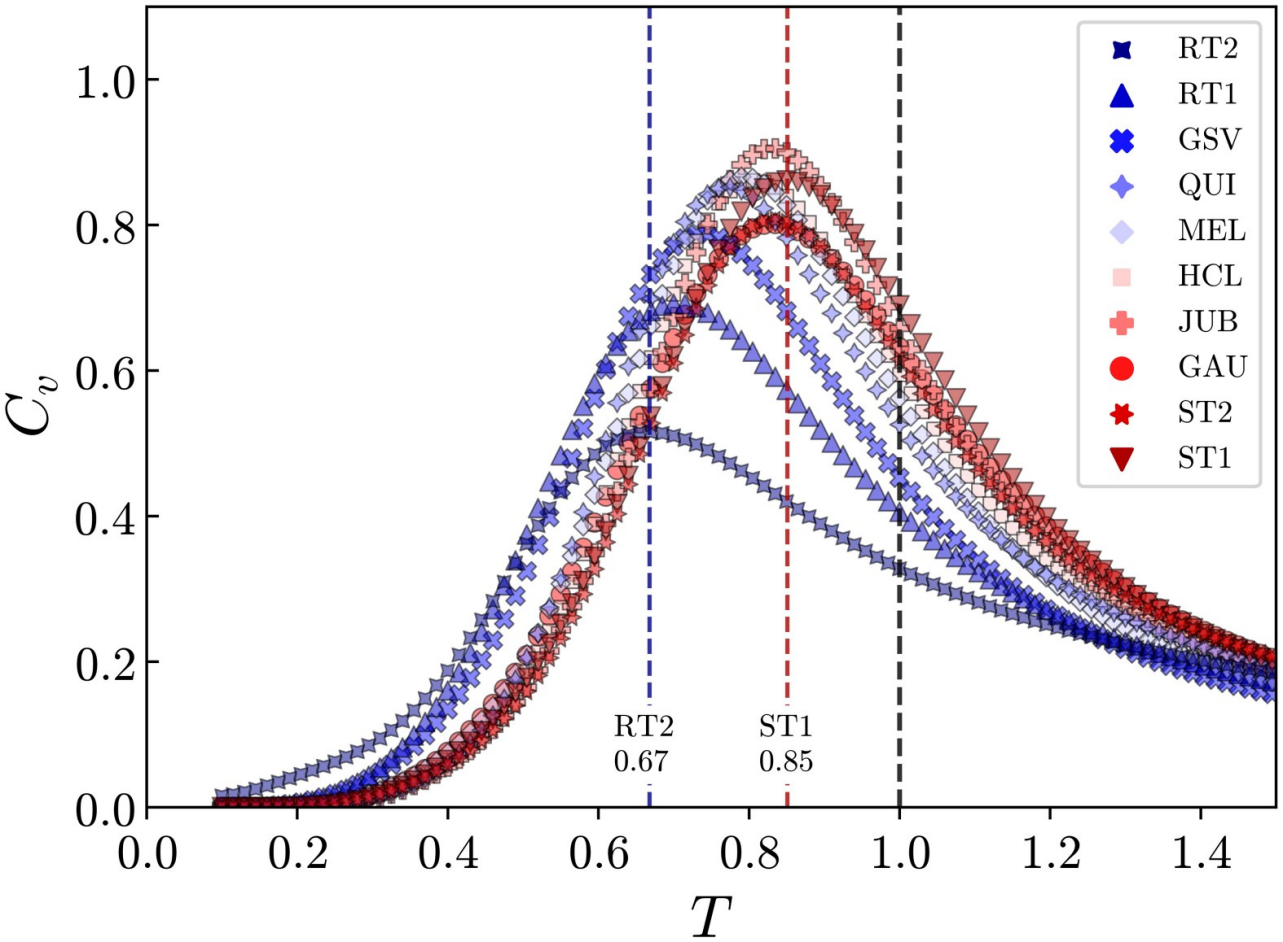
Heat capacity as a function of temperature for the system of fixation activities. Heat capacity curves for all texts, with *C*_*v*_ maximal at the critical temperature *T*_*c*_. The temperature at which the texts are being read is the operating temperature *T* = *T*_*o*_ = 1. It can be seen that the system is above and near the critical point for all texts, and the RT1 and RT2 texts are clearly the furthest.

In Fig 4 we show the variation of *C*_*v*_ as a function of *T* for all texts. As depicted, regardless of the text, the operating temperature *T*_*o*_ = 1 is always above the critical point *T*_*c*_. The distance to criticality *T*_*o*_ − *T*_*c*_, however, notably depends on the text. By simply considering the fact that larger values have been found for RT1 and RT2, as compared to the other texts (see Table 3), we can obviously anticipate that this distance can be used as an index to distinguish meaningful texts from random ones. Indeed, we will show next that *T*_*o*_ − *T*_*c*_ can be related to language processing in terms of the perceived coherence from reading a text.

**Table 3:** Distance to criticality.

Text	*T*_*o*_ − *T*_*c*_
GAU	0.169
GSV	0.262
HCL	0.192
JUB	0.170
MEL	0.207
QUI	0.229
RT1	0.296
RT2	0.332
ST1	0.149
ST2	0.167

The table reports the distances to criticality *T*_*o*_ − *T*_*c*_ calculated for different texts from eye-tracking experiments using the MEM. *T*_*o*_ = 1 is the reading operating temperature and the critical temperature *T*_*c*_ corresponds to the value of *T* where the heat capacity *C*_*v*_ for a given text is maximal.

### Survey for measuring texts complexity and coherence

#### Quantifying the complexity and coherence of the texts from a survey

In order to validate our eye-tracking results, an Internet survey was conducted by request to the MindMiners services company [51], of São Paulo, Brazil. The agency provides the usage of a digital platform that enables to develop research projects, from questionnaire creation to data collection, through a respondents panel with more than 400 thousand engaged users distributed all over Brazil (MeSeems [52]). The work methodology consists of two main stages, namely, the selection of respondents according to specific requirements of the study, and the production and revision of the questionnaire.

In our study, two groups of 200 people of diverse age, gender and place of residence were selected from the 400 thousand respondents constituting the panel of the survey agency. Details of the stratification are shown in Table 4. A minimum of high school degree was requested to ensure mature reading-comprehension skills. We did not draw a distinction between socio-economic classes. Each group of respondents was given a questionnaire, related to 5 of the total 10 texts. The texts were divided as follows:

- Group A: O Quinze, Grande Sertão: Veredas, História do Cerco de Lisboa, Story 2, Random text 2
- Group B: Jubiabá, O Gaúcho, A mão e a Luva, Story 1, Random text 1

**Table 4:** Respondents panel data.

	GroupA		GroupB	
	Respondents	Percentage	Respondents	Percentage
**Gender**				
Male	86	43.0%	90	45.0%
Female	114	57.0%	110	55.0%
**Age**				
≤ 17	2	1.0%	3	1.5%
18–24	48	24.0%	46	23.0%
25–30	26	13.0%	35	17.5%
31–40	70	35.0%	61	30.5%
≥ 41	54	27.0%	55	27.5%
**Education**				
High school	99	49.5%	83	41.5%
University	101	50.5%	117	58.5%
**Place of residence**				
Central-West	20	10.0%	20	10.0%
Northeast	46	23.0%	46	23.0%
North	10	5.0%	9	4.5%
Southeast	94	47.0%	98	49.0%
South	30	15.0%	27	13.5%

Respondents stratification based on gender, age, education level (high school degree was required) and place of residence in Brazil (indicated by regions).

The questionnaire is divided into three parts and all questions are multiple-choice. In the first part, the respondent was asked to read the texts one by one and answer a simple question related to the text content, with the purpose of merely motivating a conscious reading. In the second part, each text was presented again and the respondent was asked to rate the text complexity level in a scale of 1 to 5, ranging from a “very simple text” to a “very complex text”, respectively. Lastly, the texts were again presented and the respondent was asked to assess the text coherence level in a scale from 1 to 5, ranging from a “text not coherent at all” to a “very coherent text”, respectively. The texts were shown in a randomized order to each respondent to minimize bias in the responses.

The MindMiners company relies on manual and automatic validation of each user’s information through external data sources such as social networks and the Brazilian department of revenue. In addition, they develop algorithms to identify people with atypical behaviors such as non-compliance with questionnaire instructions and abnormal response speed. Identifiers are embedded in all of the respondent’s devices, so that the respondents panel is composed exclusively of unique users.

#### Survey main results

**Fig 5:**
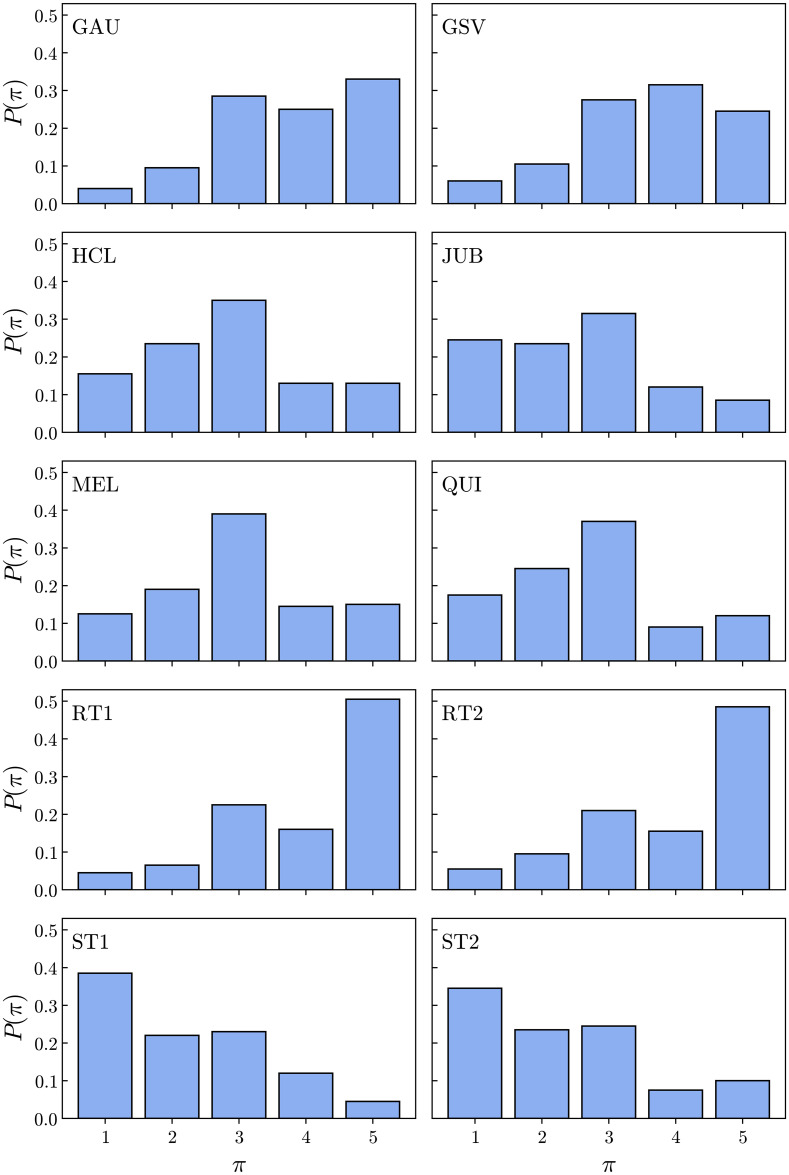
Distributions of complexity ratings. Distributions of complexity ratings among individuals for all texts read in the survey. The values *π* = 1, 2, 3, 4, 5 correspond to a scale ranging from a “very simple” text (*π* = 1) to a “very complex” text (*π* = 5).

**Fig 6:**
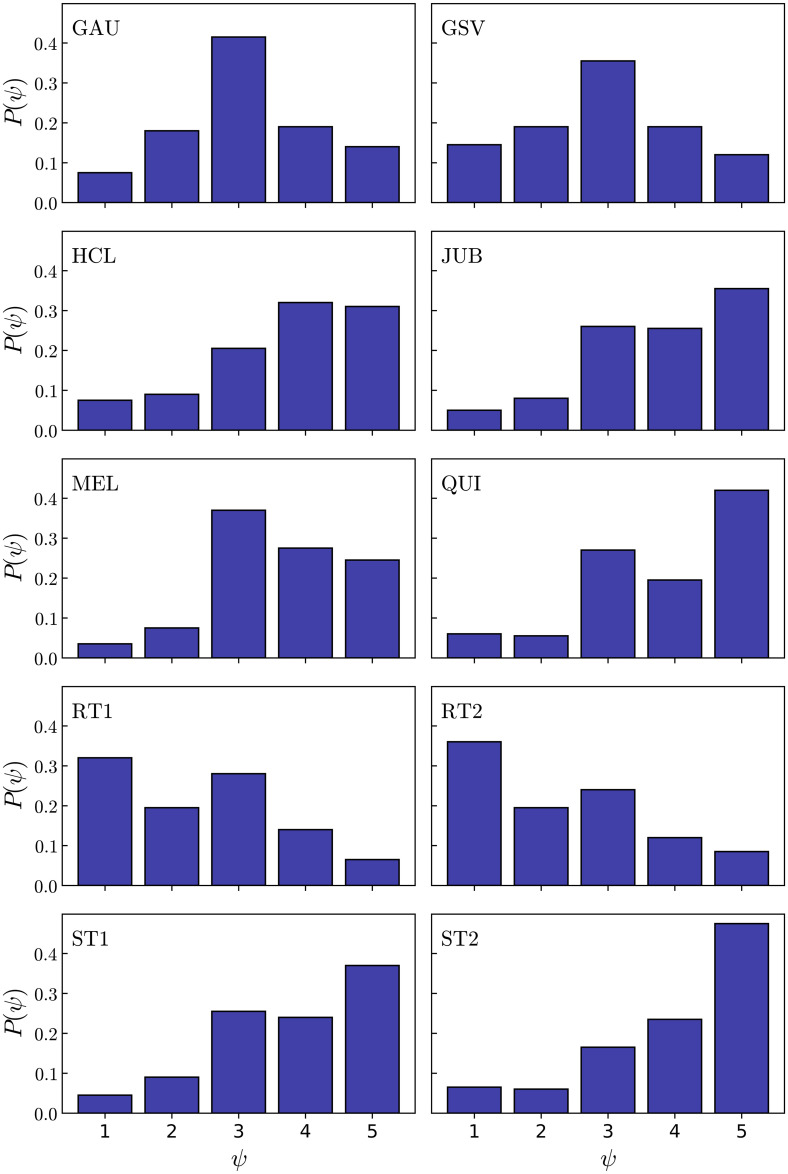
Distributions of coherence ratings. Distributions of coherence ratings among individuals for all texts read in the survey. The values *ψ* = 1, 2, 3, 4, 5 correspond to a scale ranging from a “not coherent” text (*ψ* = 1) to a “very coherent” text (*ψ* = 5).

In Fig 5 we show the distribution *P*(*π*) of fractions of individuals who rated a given value of complexity *π* for each text. For instance, we can see that almost 40% of the respondents rated the ST1 and ST2 texts as “very simple”, while up to 50% of the respondents rated the RT1 and RT2 texts as “very complex”. The other texts were rated with varied ranges of intermediate complexity values. The mean values of complexity, 〈*π*〉 are shown in Table 5. The distributions *P*(*ψ*) of fraction of individuals who rated the texts with a value *ψ* of coherence are shown in Fig 6. The results for ST1 and JUB, for example, show a very similar type of response. Most of the people (≈ 60%) ranked these texts as coherent and very coherent (grades of 4 and 5), approximately 25% ranked with an intermediate level of coherence (grade 3), and only a small fraction (less than 15%) ranked the texts with a low level of coherence (grades 1 and 2). For the random texts RT1 and RT2, on the other hand, the ratings for both are mostly in favor of a “not coherent” opinion (≈ 50%), while approximately 20% thinks that they possess an intermediate value of coherence, and little more than 15% ranked them as coherent or very coherent. Interestingly, the reading of the GSV text led to a distinctive type of response, namely, the larger group of respondents (≈ 40%) ranked the text with the intermediate grade 3. In this case, it suggests that many individuals were undecided about their evaluations on the coherence of the text.

**Table 5:** Complexity and coherence mean values.

Text	〈*π*〉	〈*ψ*〉
GAU	3.74 ± 0.08	3.14 ± 0.08
GSV	3.58 ± 0.08	2.95 ± 0.09
HCL	2.85 ± 0.09	3.70 ± 0.09
JUB	2.57 ± 0.09	3.79 ± 0.08
MEL	3.01 ± 0.09	3.62 ± 0.07
QUI	2.74 ± 0.09	3.86 ± 0.09
RT1	4.02 ± 0.08	2.44 ± 0.09
RT2	3.92 ± 0.09	2.38 ± 0.09
ST1	2.22 ± 0.09	3.80 ± 0.08
ST2	2.35 ± 0.09	4.00 ± 0.09

Complexity 〈*π*〉 and coherence 〈*ψ*〉 mean values obtained for all texts from the survey.

As shown in Table 5, the coherence mean values, 〈*ψ*〉, obtained from the survey for all texts suggest they can be sorted into three groups, namely, RT1 and RT2 have low levels of coherence (〈*ψ*〉 < 2.75), whereas the reading of ST1, ST2, JUB, HCL, MEL, QUI resulted in high coherence ratings (〈*ψ*〉 > 3.25). Finally, the GAU and GSV texts were rated with intermediate coherence levels (2.75 ≤ 〈*ψ*〉 ≤ 3.25).

## Results

### The average magnetization of the fixation activity reflects the level of text complexity

**Fig 7:**
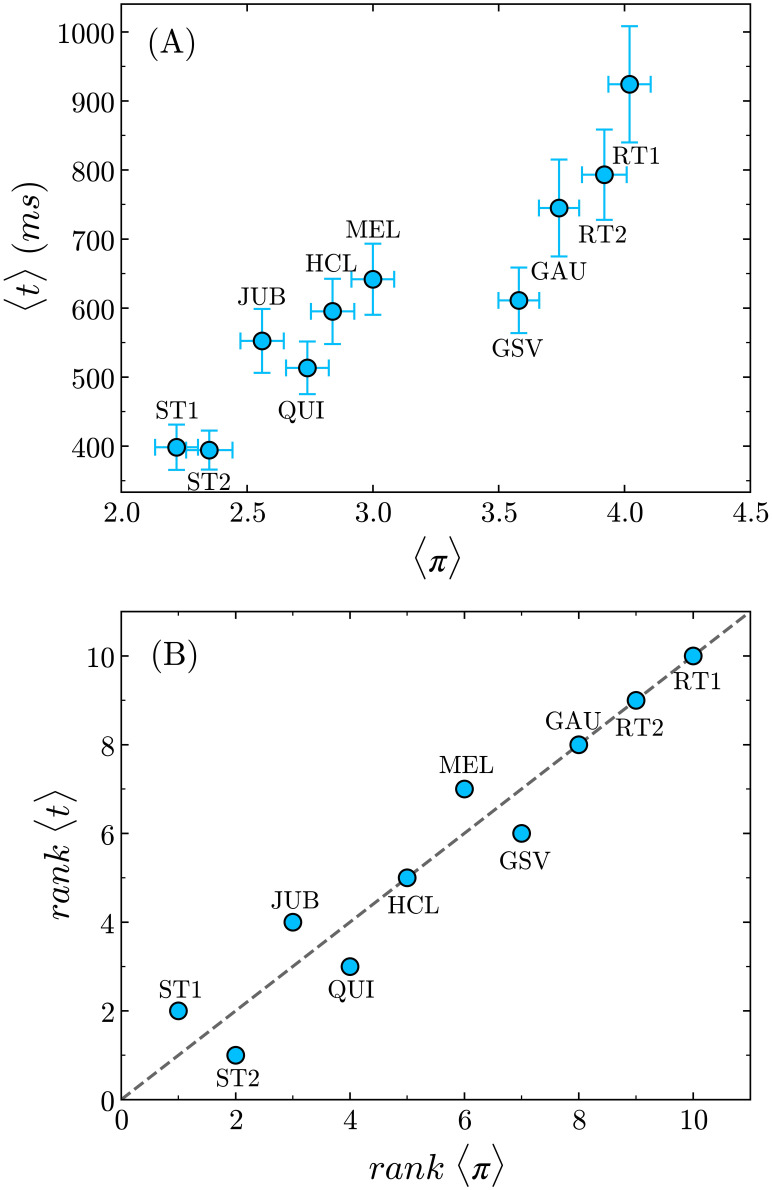
Reading times against text complexity. **(A)** The average reading time per word 〈*t*〉 generally increases with 〈*π*〉, although the relation is not monotonic. **(B)** The rank of 〈*t*〉 plotted against the rank of 〈*π*〉 shows that several discordant pairs are observed between the two variables. The dashed line corresponds to the function *y* = *x*. The Kendall rank correlation coefficient is *τ* = 0.87 (*p* = 0.0001).

As we already pointed out, the reading time span is certainly not a dispensable measure of text processing. Indeed, a correlation between reading time and the text complexity level seems then straightforward. In Fig 7(A), we plot the average reading times per word against the average values of the complexity. Although 〈*t*〉 generally increases with 〈*π*〉, the relation is hardly monotonic. This becomes more evident in Fig 7(B), where the crescent relative ranks of these variables are plotted against each other, and several discordant pairs in the rank order are observed, out of a total of ten pairs that can be compared with each other. Here we use a non-parametric statistic, namely, the Kendall rank correlation coefficient *τ*, to measure the rank correlation between 〈*t*〉 and 〈*π*〉 of the texts (see S3 Appendix). In spite of the discordant pairs, we find a value of *τ* = 0.87 (*p* = 0.0001), which indicates a reasonably high degree of correlation with positive monotonicity trend between the two variables.

**Fig 8:**
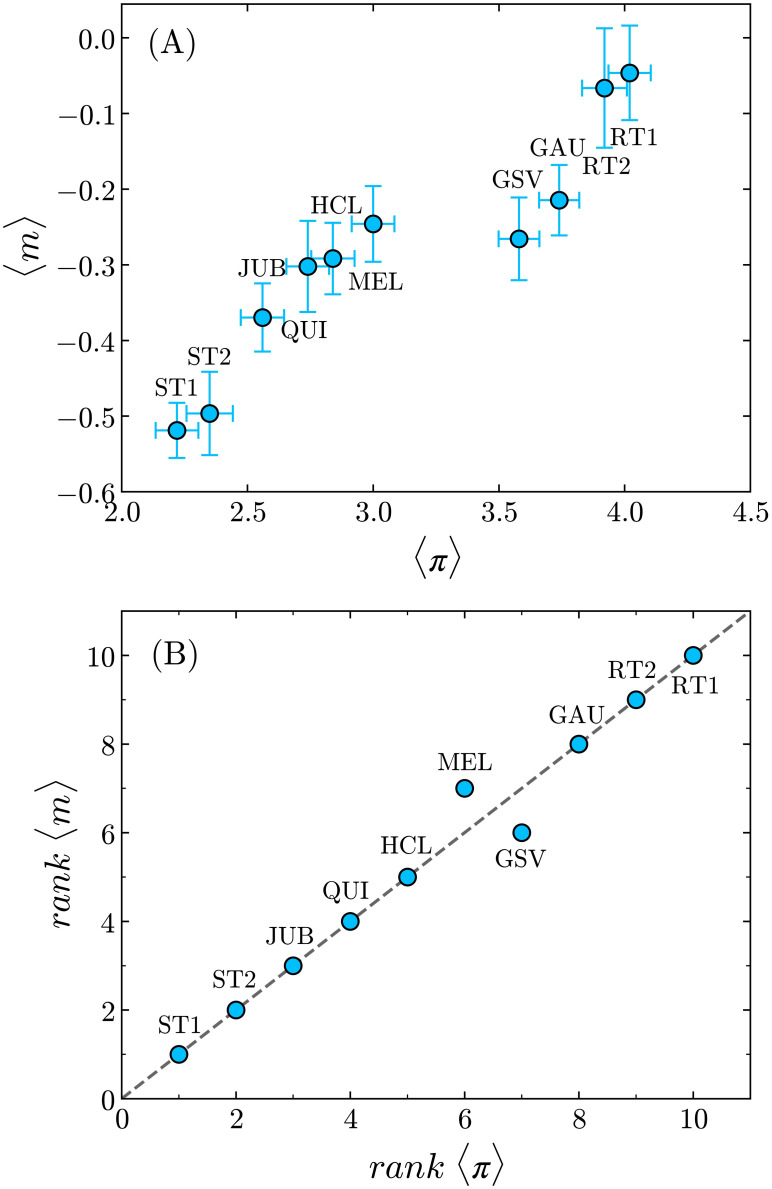
Average magnetization against text complexity. **(A)** The average magnetization, 〈*m*〉, of the fixation activities increases almost monotonically with 〈*π*〉, except for a local minimum at the complexity of GSV. **(B)** By ranking both measures in crescent order and plotting the ranks of 〈*m*〉 against the ranks of 〈*π*〉 for all texts, we can see that eight out of ten texts occupy exactly the same positions in the two lists. The dashed line corresponds to the function *y* = *x*. The Kendall rank correlation coefficient *τ* = 0.96 (*p* = 5 × 10^−6^) indicates the very high trend of monotonicity between the two variables.

The situation is rather different when we plot the average magnetization, 〈*m*〉, against 〈*π*〉 for all texts, as shown in Fig 8(A). The two measures are highly correlated, although in a nonlinear fashion, with 〈*m*〉 increasing almost monotonically with 〈*π*〉, except for a capricious local minimum at the average complexity of GSV. Moreover, by plotting the relative ranks of 〈*m*〉 and 〈*π*〉 for a given text against each other (see Fig 8(B)), we notice that, out of ten texts, eight of them occupy identical positions in both lists. As compared to the reading time per word, the higher Kendall rank correlation coefficient found in this case, *τ* = 0.96 (*p* = 5 × 10^−6^), confirms that the measure 〈*m*〉 certainly represents a better proxy to rank the complexities 〈*π*〉 of the texts.

One may argue that more complex texts are expected to require more time for analysis, and therefore more fixations overall. However, when considering the average reading times per word, we observe that, for example, it takes around 80 more milliseconds per word on average to read the HCL text than to read the QUI text (a 15% difference), in spite of their similar levels of complexity, according to the survey. The same happens with the GSV and GAU texts, with the subjects spending an additional 133 milliseconds per word on average to read the latter (a difference of 20%), even though their relative difference in average complexities is smaller than 5% (see Table 5). In this regard, a comparison between Figs 7(B) and 8(B), and between their corresponding Kendall coefficients, unambiguously indicate that the average magnetization represents a more reliable indicator of the perceived complexity than the reading time per word.

### Text coherence perception evidenced by distance to criticality

**Fig 9:**
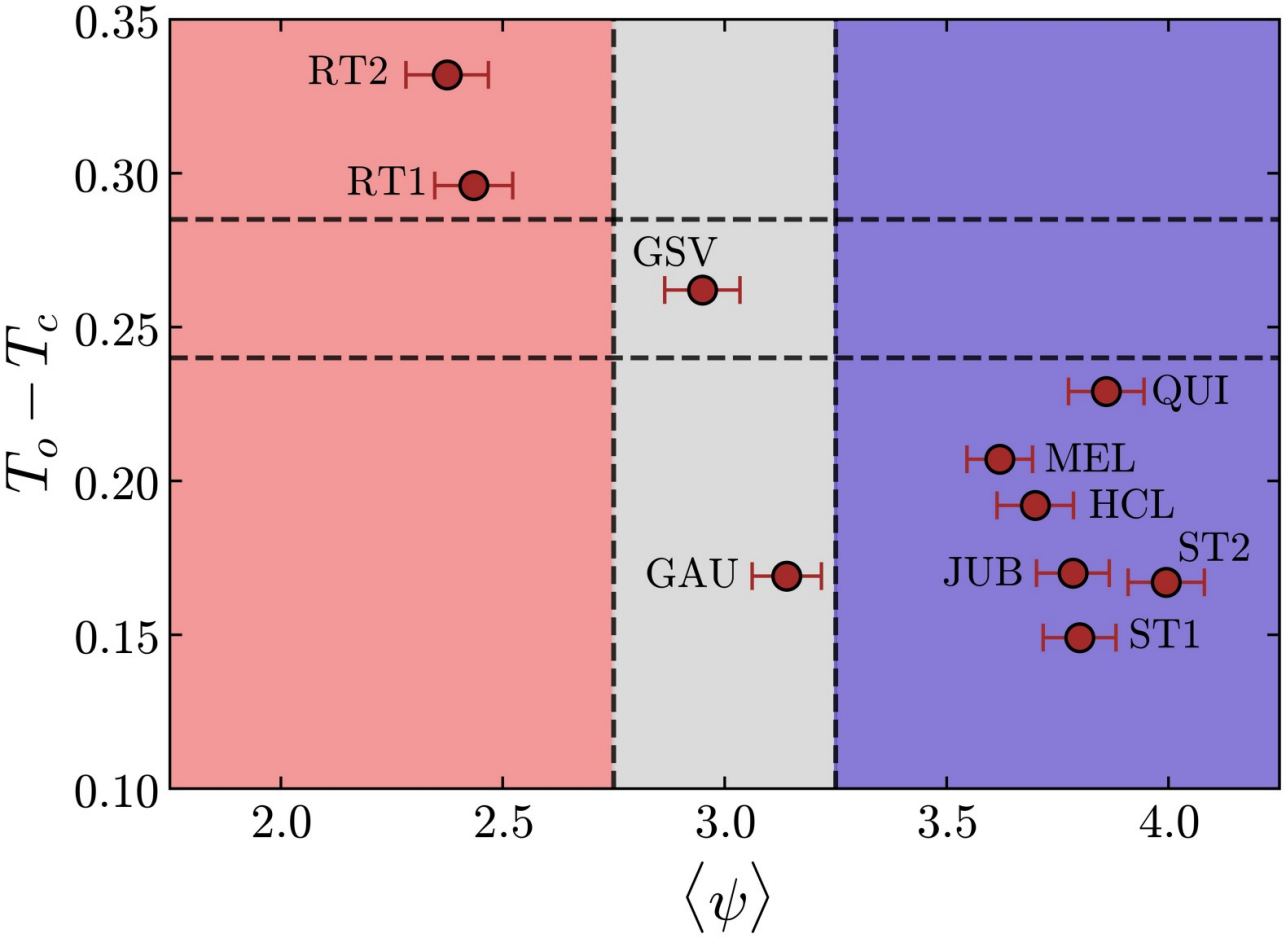
Distance to criticality and text coherence. Relation between the distance to criticality *T*_*o*_ − *T*_*c*_ and the average coherence 〈*ψ*〉 of the texts. Texts rated with low coherence 〈*ψ*〉 < 2.75 are associated with large values of *T*_*o*_ − *T*_*c*_ (RT1 and RT2), while texts considered to be coherent 〈*ψ*〉 > 3.25 are close to criticality (ST1, ST2, JUB, HCL, MEL, QUI), suggesting an implicit cohesive reading response among individuals. The two texts rated with intermediate values of 〈*ψ*〉 (GAU and GSV), however, induced rather distinct responses in terms of *T*_*o*_ − *T*_*c*_.

The results shown in Fig 9 reveal that large distances to criticality (*T*_*o*_ − *T*_*c*_) are consistent with the low coherent nature (〈*ψ*〉 < 2.75) of both random texts (RT1 and RT2). Moreover, all texts rated with high coherence 〈*ψ*〉 > 3.25 (ST1, ST2, JUB, HCL, MEL, and QUI) group at the bottom-left corner of the plot due to their correspondingly small values of (*T*_*o*_ − *T*_*c*_). Coherent texts therefore prompt higher correlated responses in the fixation activity of the readers, suggesting implicit cohesive interactions among them. Interestingly, the two texts ranked with intermediate values of coherence 2.75 < 〈*ψ*〉 < 3.25 in the survey (GAU and GSV), however, induced very different responses in terms of the distance to criticality obtained from the eye-tracked readings. In order to understand this difference, a more detailed analysis is required with respect to the literary styles and linguistic aspects of the books from where these texts have been extracted, as we present in the Discussion.

It is important to stress that one can only rely on the particular features of the cross-correlations from the fixation activity series (see Fig 3) between pairs of readers for a given text to justify the clear numerical differences found among the values of (*T*_*o*_ − *T*_*c*_). In order to test for this hypothesis, we perform additional calculations with the fixation activities of the subjects for a given text, preserving the mean magnetization 〈*σ*_*i*_〉, but shuffling the values of *σ*_*i*_ among randomly chosen pairs of words in the text. In this way, strong correlations, if present between the fixation activities, should disappear. Once we have shuffled the data, we follow the same sequence of calculations as before, namely, we find the pairwise correlations *C*_*ij*_, compute the fields *h*_*i*_ and couplings *J*_*ij*_, and determine the heat capacity *C*_*v*_ at different temperatures *T*. The effect of suppressing strong correlations is to substantially reduce the interactions *J*_*ij*_, therefore decreasing the value of *T*_*c*_ for all texts and increasing their distance (*T*_*o*_ − *T*_*c*_) (see S2 Appendix).

## Discussion

Linguists have long studied the notions of text complexity and coherence. More recently, attempts to measure these quantities have been made through mathematical expressions, known as readability formulas, that mainly rely in metrics of word and sentence lengths and word frequency. However, it is arguable whether these formulas are sufficient or not to determine the complexity of a text, mostly for two sound reasons. First, there are uncomplicated pieces of writing that use many infrequent words (an indicator of high complexity for these formulas), like informational texts, as the one suggested in [25], “Any text on raccoons would use ‘raccoon’ a lot, as well as ‘nocturnal’ and ‘foraging’”. This is a typical example for which most readability expressions would overrate the complexity score of the text. Second, texts encompassing elaborated ideas can nevertheless be written with words from a simple vocabulary and constituted of short sentences. This is the case, for example, of some texts containing abstract narratives, usage of metaphors and obscure allusions, for which those complexity scores would be underrated. A well-known example in the literature is Ernest Hemingway’s book “The Old Man and the Sea” that could be easily underrated in complexity by readability formulas, despite its profoundness and story-rich writing. As a consequence, in addition to using these metrics, a qualitative analysis is usually recommended by linguists in order to more adequately categorize the reading material.

Here, instead of applying empirical mathematical expressions, we approached the problem of quantifying the complexity and coherence of texts by using data from an extensive survey with a group of 400 people, and comparing it with properties from reading patterns obtained with eye-tracking experiments. We calculated the magnetizations, the most elemental measure of our system that represents the density of fixation activity that the readers had for each text. We hypothesized and confirmed with the survey that the more complex the text the greater the average magnetization. These results therefore suggest that the fixation acitivity, as we defined here, contains the sufficient cognitive information to characterize the reading patterns in terms of active and inactive states. The adopted threshold for the number of fixations in a given word *r* ($n_{i}^{r}=2$) succeeded in delimiting the minimum to characterize the words in the text that need further cognitive processing. Furthermore, it somehow accounts for the effect of repetitive fixation due to word size and low frequency, while relativizing the underlying variations among the fixation patterns of individuals related to their particular reading skills and capability to predict the occurrence of words in context. This was verified by testing larger values of threshold. Already for a threshold of 3, the fixation activities become heavily dominated by the words size and/or their frequency, showing significant dissimilarities among subjects. In this framework, the threshold adopted in our work acts as a simple, but surprisingly effective way to somehow attenuate differences among subjects.

The fact that the randomly generated texts (RT1 and RT2) were rated with low levels of coherence, while most of literary texts were considered to be coherent by the readers in the survey could be anticipated. The intermediate ratings of coherence for the literary texts GSV and GAU, however, are certainly worth of a more detailed analysis. The celebrated Brazilian author, Guimarães Rosa, who wrote “Grande Sertão: Veredas”, from which the fragment GSV was extracted, is well-known for his distinctive writing style, frequently compared to that of James Joyce, in what concerns to the astonishing linguistic work and experimentation [53].

In the literary work of Rosa, we often find unconventional punctuation and grammar in story-rich writing, while creating neologisms from erudite and popular expressions, regionalisms, archaic words and inventive use of prefixes and suffixes [53, 54]. In fact, many of these linguistic features are found in the GSV excerpt used here, with which the book opens. The first word of the text, “nonada” is already an unusual term that, although existing in the Portuguese language, is old-fashioned and hardly used in literature. Even in context, the expression seems so enigmatic that it has led to different interpretations over the years [55–57]. The second sentence has a non-traditional syntactic structure, typical of regional orality, as we learn from the study of Garcia [58]. We see that this linguistic resource is found in the fifth and fifteenth sentences as well. The author also makes use of incomplete suggestive expressions in three sentences of the text (second, fifth and seventh sentences), a linguistic construction typical of Rosa’s writing. In addition to this, there are two neologisms in the text created by the author, namely, “erroso” and “prascovio” (in the eighth and thirteenth sentences, respectively). Without entering into a denser analysis, it is fair to say that the GSV fragment is not part of a conventional literary work, being quite difficult to grasp, especially when removed from the global context of the book’s narrative. We therefore conjecture that the reader might feel confused and undecided when processing the text, finding it hard to qualify the narrative as coherent.

The text GAU, on its turn, is a transcription from the novel “O Gaúcho”, written by José de Alencar in the year 1870. The fragment was extracted from the end of chapter one, where the setting in which the story takes place is described. The writing is characterized by an overwhelming, philosophical representation of the scenario [59]. The abstract tone in the narrative possibly gives the reader an impression that the text is somehow vague, leading to the uncertainty in qualifying it as coherent. In direct contrast with GSV and GAU, the other excerpts of literary works employed here correspond to passages of descriptive, straightforward writings (HCL, MEL), or linear, plain storytelling (QUI, JUB, ST1, ST2), which very likely make them easy to interpret and therefore be considered as coherent.

Our results from a very simple statistical model and from the analysis with the Pairwise Maximum-Entropy method revealed that the distance to the critical point was capable to segregate the texts into three main groups. The random generated texts (RT1 and RT2) are the farthest from the critical point, with the operating temperatures *T*_*o*_ significantly higher than *T*_*c*_, the GSV text follows just behind, and the rest of the texts fall much closer to *T*_*c*_. As we argued previously, in the physical context of critical phenomena, when *T*_*o*_ > *T*_*c*_, the interactions between the component elements are weak and the system is in a disordered state. Our results then suggest that the fixation activities for texts with low coherence (RT1 and RT2) are random to a certain degree, meaning that the reading response to the text stimuli does not promote strong virtual connections among different individuals. When *T*_*o*_ approaches *T*_*c*_, the “interactions” among elements increase and local effects can propagate over the entire group. The system then becomes susceptible to global changes, and a collective behavior may emerge. This effect has been observed in the texts that were rated with high levels of coherence (ST1, ST2, JUB, HCL, MEL, and QUI) and also with the GAU text, although it was rated with intermediate average coherence. We reason that a high degree of coherence in a text is likely to induce a cohesive reading response, here manifested in terms of a proximity to its critical point. Although the readers never interact with one another, we can think of them responding with a similar cognitive behavior when the content of the text is consistent. A question that naturally arises is why the relation between the average coherence rating 〈*ψ*〉 and *T*_*o*_ − *T*_*c*_ is ambiguous for the GAU and GSV texts, given that the former falls into the cluster of texts with operating temperatures close to *T*_*c*_, while the latter is far from *T*_*c*_, and still both of them were rated on average with intermediate levels of coherence. Previously we elaborated on the characteristics of these texts, and referred to the linguistic features that made them stand out from the other literary fragments investigated here. On the case of the GAU excerpt, the reader has to process an intelligible text, and can yet have a dubious interpretation due to the abstract style of the writing. Perhaps rating this type of text with a specific value of coherence is equivocal, but we can fairly state that the content of the text is coherent, *i.e*., its narrative is consistent. In contrast, the GSV text appears atypical to an average reader because of its highly technical writing and uncommon linguistic elements. In a sense, we can think of the GSV as an intermediate type of text, in between a concrete narrative and a random incongruous one. Taking this and the fact that GAU otherwise induced a low value for *T*_*o*_ − *T*_*c*_, the results shown in Fig 9 evidence that the distance to the critical point is actually segregating the texts according to some coherence measure. Such a measure, which originates from an inner cognitive mechanism, is perhaps less subjected to the influence of extrinsic factors than the response to a questionnaire within the protocol of a digital survey.

## Conclusion

In summary, the results presented here show that eye-tracking data can be duly processed and analyzed to produce consistent proxies for complexity and coherence of diverse texts. The same texts, including children stories, random word generated texts and excerpts from literature work, have been used to validate this hypothesis by means of an extensive Internet survey with a large number of readers. Our results were substantiated by (*i*) the nearly monotonic relation between the average magnetization 〈*m*〉 of the fixation activities and the average complexity 〈*π*〉 of the texts and (*ii*) the suitability of the distance (*T*_*o*_ − *T*_*c*_) to segregating random texts (meaningless in content, but with preserved grammar structures) from coherent ones. We recall that the curve *T* − *T*_*c*_ for each text is computed from the “energy” of the system, which we obtain by applying the maximum-entropy learning algorithm to the fixation activities of all eye-tracked readers. This finding is particularly significant for several reasons. For one thing, it is another example of how learning algorithms are efficient in extracting relevant information out of large amounts of experimental data, and specifically it supports the maximum-entropy approach as an elementary yet solid method to study complex systems. At the same time, we get to notice how humans respond cohesively to a coherent, consistent text, which is indicative of the advanced language formation and reading prediction mechanisms that we have developed. Instead, when the written information is nonsensical, the collective cognitive response is dispersed.

## Supporting information

S1 Appendix Derivation of the Maximum-Entropy Model and inverse Ising problem. (PDF)

S2 Appendix Randomization of fixation activities. (PDF)

S3 Appendix Kendall correlation coefficient. (PDF)

S4 Appendix Survey raw data. (PDF)

S1 Fig Experimental values of magnetizations against theoretical values. The calculated values 〈*σ*_*i*_〉_*th*_ reproduce the experimental values 〈*σ*_*i*_〉, as evidenced by the plots falling on the red dashed lines corresponding to *y* = *x*.
(JPG)

S2 Fig Experimental values of covariances against theoretical values. The calculated values 〈*σ*_*i*_*σ*_*j*_〉_*th*_ reproduce the experimental values 〈*σ*_*i*_*σ*_*j*_〉, as evidenced by the plots falling on the red dashed lines corresponding to *y* = *x*.
(JPG)

S3 Fig Heat capacity as a function of temperature for the system of fixation activities with shuffled data. Average heat capacity curves for all texts, after shuffling the values of fixation states *σ*_*i*_ among randomly chosen pairs of words in the text. The average values are calculated over 100 shuffling trials and the error bars are smaller than the symbols. This suppresses strong correlations, here evidenced by a a significant increase of the distance to the critical point (*T*_*o*_ − *T*_*c*_).
(JPG)

S4 Fig Distance to criticality and text coherence with shuffled data. Relation between the average distance to criticality 〈*T*_*o*_ − *T*_*c*_〉 and the average coherence 〈*ψ*〉 of the texts, after shuffling the values of fixation states *σ*_*i*_ among randomly chosen pairs of words in the text.
(JPG)

S1 Table Distance to criticality with shuffled data. The table reports the average distances to criticality 〈*T*_*o*_ − *T*_*c*_〉 calculated using the MEM by shuffling the data from the fixation maps of the eye-tracking experiments (average calculated over 100 trials). *T*_*o*_ = 1 is the reading operating temperature and the critical temperature *T*_*c*_ corresponds to the value of *T* where the heat capacity *C*_*v*_ for a given text is maximal.
(PDF)

S2 Table Group A and Group B survey raw data. (PDF)
