# Supplementary material for: Eye-tracking as a proxy for coherence and complexity of texts
Source: PLoS One. 2021 Dec 13;16(12):e0260236. doi: 10.1371/journal.pone.0260236 (PMC8668102; doi:10.1371/journal.pone.0260236)
Supplement: S1 Appendix — (PDF) [file pone.0260236.s001.pdf]

S1 Appendix. Derivation of the Maximum-Entropy Model and inverse Ising problem.

Let it be P the probability distribution that represents the state of the system $\sigma = \{\sigma_1^r, ..., \sigma_N^r\}$ for the N subjects at word r. We derive P following the Maximum Entropy principle, conditional on reproducing the experimental values of magnetization and covariances for all the subjects.

The first requirement is that P be normalized,

$$\sum_{\{\sigma\}} P(\{\sigma\}) = 1 \tag{1}$$

Then, we demand that the first moment of P matches the sample average, for $i = 1, ..., N$,

$$\langle \sigma_i \rangle_P = \sum_{\{\sigma\}} P(\{\sigma\})\sigma_i = \langle \sigma_i \rangle = \frac{1}{M}\sum_{r=1}^{M} \sigma_i^r \tag{2}$$

and the second moment of P equals the measured covariances values, for $i, j = 1, ..., N$, $i \neq j$,

$$\langle \sigma_i \sigma_j \rangle_P = \sum_{\{\sigma\}} P(\{\sigma\})\sigma_i\sigma_j = \langle \sigma_i\sigma_j \rangle = \frac{1}{M}\sum_{r=1}^{M} \sigma_i^r\sigma_j^r \tag{3}$$

Finally, we ask that $P(\{\sigma\})$ maximizes the entropy,

$$S = -\sum_{\{\sigma\}} P(\{\sigma\}) \ln\, P(\{\sigma\}) \tag{4}$$

This problem is solved using the method of Lagrange multipliers, *i.e*, we look for P such that S is maximal subject to the equality constraints given by Eqs. (1)-(3). The Lagrangian for this problem is of the form,

$$\mathcal{L} = S + \lambda_0(\langle 1 \rangle_P - 1) + \sum_{i=1}^{N} \lambda_{1_i}(\langle \sigma_i \rangle_P - \langle \sigma_i \rangle) + \sum_{i,j=1}^{N} \lambda_{2_{ij}}(\langle \sigma_i\sigma_j \rangle_P - \langle \sigma_i\sigma_j \rangle) \tag{5}$$

and must satisfy,

$$\frac{\partial \mathcal{L}}{\partial P(\{\sigma\})} = 0 \;\Rightarrow\; -\ln\, P(\{\sigma\}) - 1 + \lambda_0 + \sum_{i=1}^{N} \lambda_{1_i}\sigma_i + \sum_{i,j=1}^{N} \lambda_{2_{ij}}\sigma_i\sigma_j = 0 \tag{6}$$

where λ_0, $\{\lambda_{1_i}\}$ and $\{\lambda_{2_{i,j}}\}$ represent the Lagrange multipliers. The solution for Eq. (6) is,

$$P(\{\sigma\}) = \frac{1}{Z}\exp\left(\sum_{i=1}^{N} \lambda_{1_i}\sigma_i + \sum_{i,j=1}^{N} \lambda_{2_{ij}}\sigma_i\sigma_j\right) \tag{7}$$

where Z is the partition function that represents the normalization constant,

$$Z = \sum_{\{\sigma\}} \exp\left(\sum_{i=1}^{N} \lambda_{1_i}\sigma_i + \sum_{i,j=1}^{N} \lambda_{2_{ij}}\sigma_i\sigma_j\right) = \exp\left(1 - \lambda_0\right) \tag{8}$$

From Eq. (7) we see that for a system of pairwise interacting particles, P is equal to the Boltzmann's probability distribution at temperature $T = 1$. Furthermore, the energy term corresponds to the random field Ising Hamiltonian [1], with $\lambda_{1_i} = h_i$ and $\lambda_{2_{ij}} = J_{ij}$.

The inverse Ising problem consists in finding the unknown couplings J_{ij} and fields h_i from the known observables $\langle \sigma_i \sigma_j \rangle$ and $\langle \sigma_i \rangle$. From Eq. 8, it follows that

$$\langle \sigma_i \rangle = \frac{\partial}{\partial h_i} \ln Z(\mathbf{J}, \mathbf{h}) \quad (9)$$

$$\langle \sigma_i \sigma_j \rangle = \frac{\partial}{\partial J_{ij}} \ln Z(\mathbf{J}, \mathbf{h}) \quad (10)$$

where $\mathbf{J} = \{J_{ij}\}$ and $\mathbf{h} = \{h_i\}$. By solving Eqs. 9 and 10 we are able to infer the couplings and fields for our system, as we show in S1 Fig and S2 Fig. It can be seen that the obtained theoretical values $\langle \sigma_i \rangle_{th}$ and $\langle \sigma_i \sigma_j \rangle_{th}$ reproduce the experimental values to high precision.

By knowing the probability distribution of the system, other quantities can be estimated. The heat capacity C_v, defined as the rate in which the average energy of the system changes with the temperature, is calculated here in terms of the mean square deviation of the energy [2]:

$$C_v = \frac{\beta}{N} \langle E^2 \rangle - \langle E \rangle^2 \quad (11)$$

by means of the partition function. Then, the critical temperature is determined as the point in which C_v is maximal.

References

1. Belanger DP, Young AP. The random field Ising model. Journal of Magnetism and Magnetic Materials. 1991;100(1):272–291. doi:https://doi.org/10.1016/0304-8853(91)90825-U.

2. Newman MEJ, Barkema GT. 1-4. In: Monte Carlo methods in statistical physics. Oxford: Clarendon Press; 1999.
