# Supplementary material for: Eye-tracking as a proxy for coherence and complexity of texts
Source: PLoS One. 2021 Dec 13;16(12):e0260236. doi: 10.1371/journal.pone.0260236 (PMC8668102; doi:10.1371/journal.pone.0260236)
Supplement: S2 Appendix — (PDF) [file pone.0260236.s002.pdf]

S2 Appendix. Randomization of fixation activities.

S3 Fig shows the variation of the heat capacity C_v on the temperature T for each text after shuffling the data in the lines of their corresponding fixation maps. We performed 100 shuffling trials and calculated the average temperatures and heat capacities for every text. In this case, the average distance to criticality, $\langle T_o - T_c \rangle$, notably increases for all texts, evidencing that the removal of significant correlations present in the original data (without shuffling) prevents the segregation of texts in terms of their coherence level (see S4 Fig). In S1 Table, we show the actual values of $\langle T_o - T_c \rangle$ for this case.
