# Supplementary material for: Eye-tracking as a proxy for coherence and complexity of texts
Source: PLoS One. 2021 Dec 13;16(12):e0260236. doi: 10.1371/journal.pone.0260236 (PMC8668102; doi:10.1371/journal.pone.0260236)
Supplement: S3 Appendix — (PDF) [file pone.0260236.s003.pdf]

S3 Appendix. Kendall correlation coefficient.

The Kendall rank correlation coefficient τ is a non-parametric statistic used to measure the rank correlation between two quantities, *i.e.*, the degree of correspondence of their rankings [1]. Precisely, let $(x_1, y_1), \ldots, x_n, y_n)$ be a set of observations of the joint variables X and Y. A pair (x_i, y_i) and (x_j, y_j) are concordant if the sort order of (x_i, x_j) and (y_i, y_j) are compatible, namely, if either both $x_i > x_j$ and $y_i > y_j$ or both $x_i < x_j$ and $y_i < y_j$ hold, otherwise they are discordant. The Kendall coefficient is defined as,

$$\tau = \frac{2}{n(n-1)} \sum_{i<j} sgn(x_i - x_j)\ sgn(y_i - yj)$$

where *sgn* represents the sign function. Values of τ close to 1 indicate strong agreement between the two quantities, and values close to -1 indicate strong disagreement. We calculate the p-value for a hypothesis test which null hypothesis is an absence of association, that is, $\tau = 0$.

References

1. Kendall MG. A New Measure of Rank Correlation. Biometrika. 1938;30(1/2):81–93.
