# Supplementary material for: Eye-tracking as a proxy for coherence and complexity of texts
Source: PLoS One. 2021 Dec 13;16(12):e0260236. doi: 10.1371/journal.pone.0260236 (PMC8668102; doi:10.1371/journal.pone.0260236)
Supplement: S4 Appendix — (PDF) [file pone.0260236.s004.pdf]

S4 Appendix. Survey raw data.

In S2 Table we show the survey data, specifying the stratification details for all respondents, duly de-identified, as well as their individual answers to the questionnaire. The stratification columns include information of the respondents': survey ID number, date of the survey, gender, age, city, state, region and education. This information is summarized in Table 4 of the main text. The two tables correspond to the respondents groups A and B, as we explain in the main text. All the data was provided by the MindMinders company.
