# Supplementary figures and images for: Eye-tracking as a proxy for coherence and complexity of texts
Source: PLoS One. 2021 Dec 13;16(12):e0260236. doi: 10.1371/journal.pone.0260236 (PMC8668102; doi:10.1371/journal.pone.0260236)

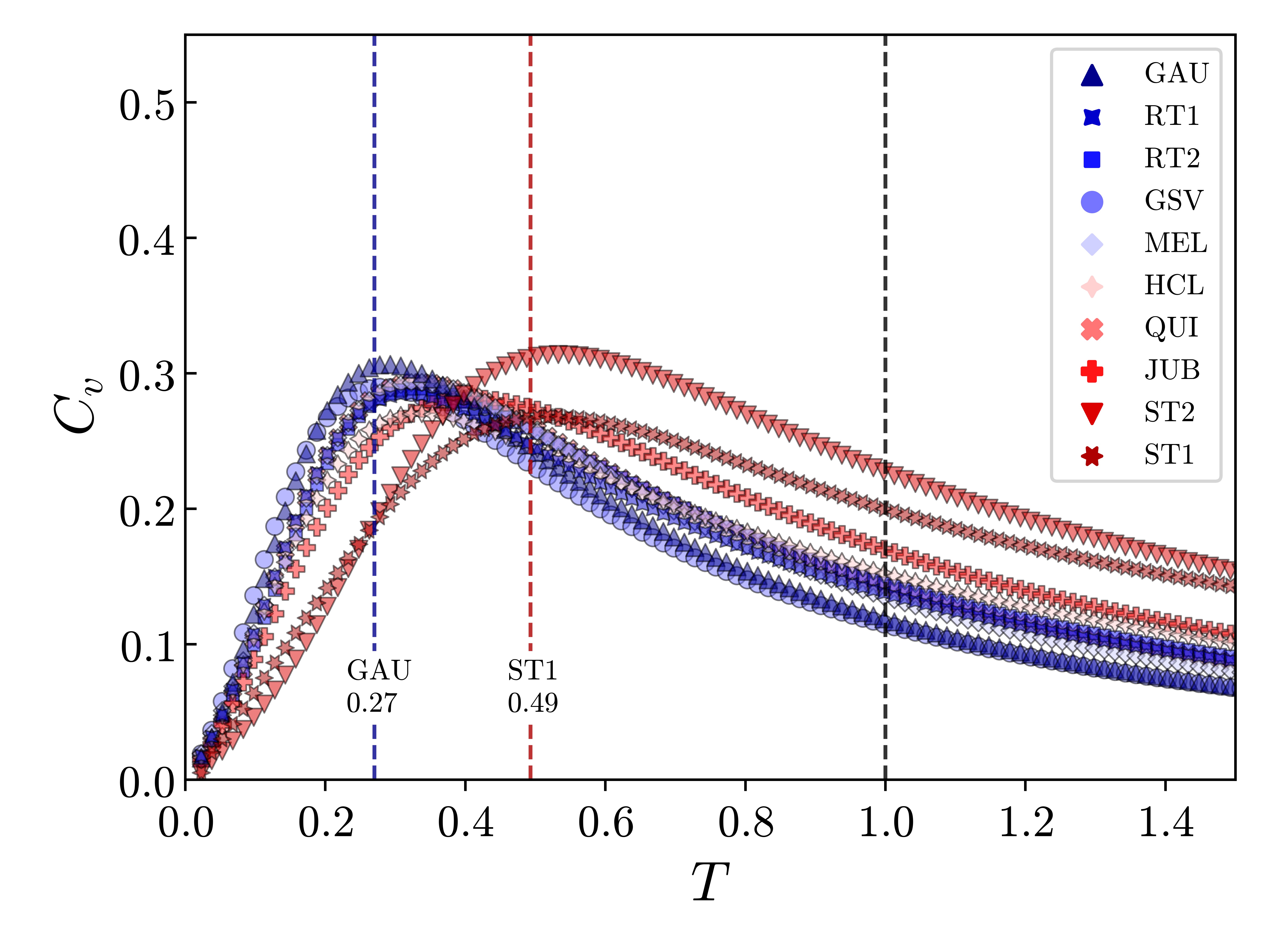

Supplement: S3 Fig — Average heat capacity curves for all texts, after shuffling the values of fixation states σi among randomly chosen pairs of words in the text. The average values are calculated over 100 shuffling trials and the error bars are smaller than the symbols. This suppresses strong correlations, here evidenced by a a significant increase of the distance to the critical point (To − Tc). (JPG) [file pone.0260236.s007.jpg]

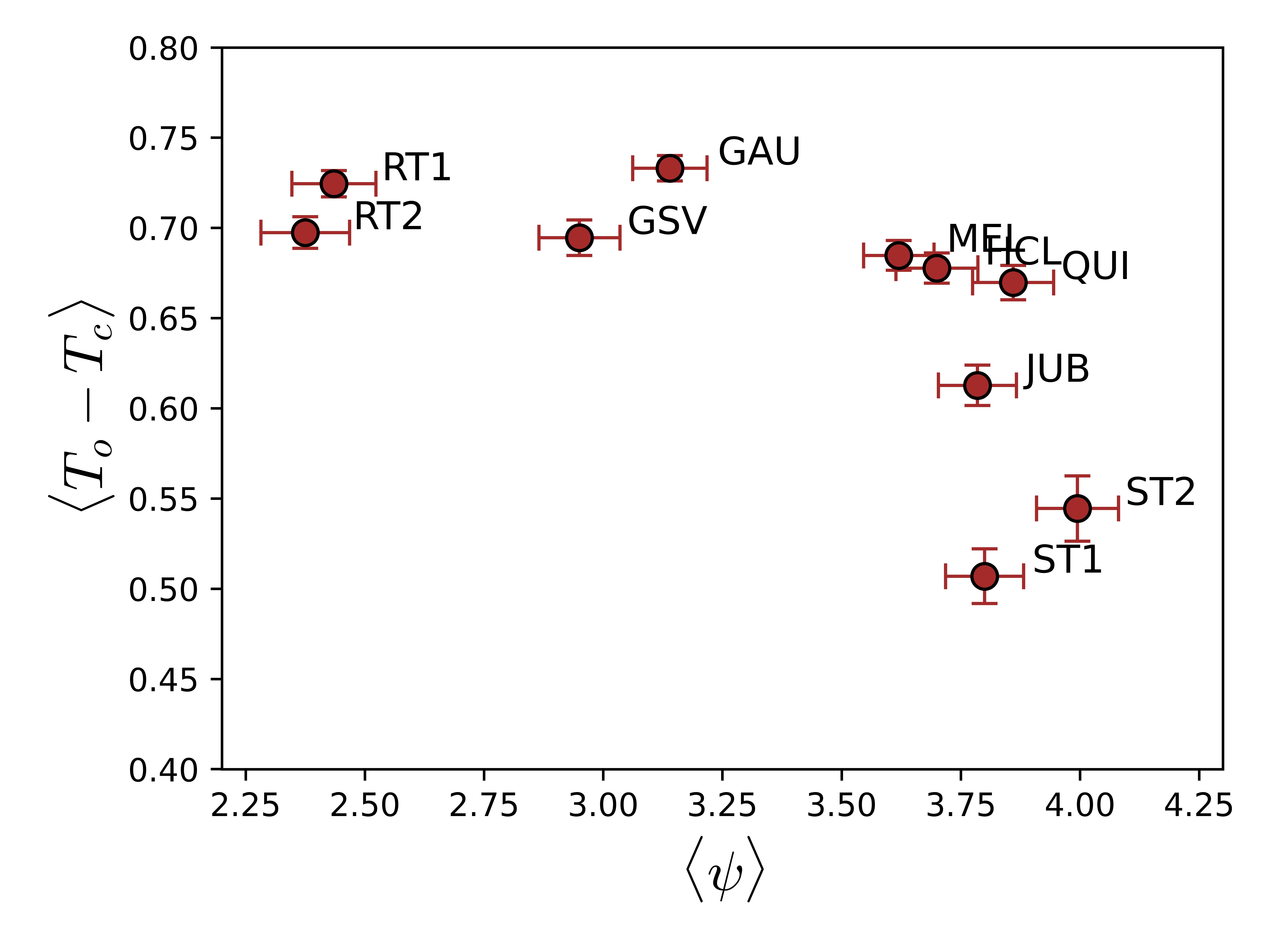

Supplement: S4 Fig — Relation between the average distance to criticality 〈To − Tc〉 and the average coherence 〈ψ〉 of the texts, after shuffling the values of fixation states σi among randomly chosen pairs of words in the text. (JPG) [file pone.0260236.s008.jpg]
