# Supplementary material for: Eye-tracking as a proxy for coherence and complexity of texts
Source: PLoS One. 2021 Dec 13;16(12):e0260236. doi: 10.1371/journal.pone.0260236 (PMC8668102; doi:10.1371/journal.pone.0260236)
Supplement: S1 Table — The table reports the average distances to criticality 〈To − Tc〉 calculated using the MEM by shuffling the data from the fixation maps of the eye-tracking experiments (average calculated over 100 trials). To = 1 is the reading operating temperature and the critical temperature Tc corresponds to the value of T where the heat capacity Cv for a given text is maximal. (PDF) [file pone.0260236.s009.pdf]

Table 1. Distance to criticality with shuffled data.

**Text**	$\langle T_o - T_c \rangle$ **(with shuffling)**
GAU	0.733 ± 0.007
GSV	0.695 ± 0.010
HCL	0.678 ± 0.008
JUB	0.613 ± 0.010
MEL	0.685 ± 0.008
QUI	0.670 ± 0.010
RT1	0.724 ± 0.007
RT2	0.697 ± 0.009
ST1	0.507 ± 0.015
ST2	0.544 ± 0.018
