# Supplementary material for: Eye-tracking as a proxy for coherence and complexity of texts
Source: PLoS One. 2021 Dec 13;16(12):e0260236. doi: 10.1371/journal.pone.0260236 (PMC8668102; doi:10.1371/journal.pone.0260236)
Supplement: S2 Table — (PDF) [file pone.0260236.s010.pdf]

Group A

User ID	Data	Gênero	Idade	Cidade	Estado	Região	Escolaridade	QUI- Em uma escala de um a cinco, onde 1 é muito complexo e 5 é muito simples, como você classificaria a complexidade do texto que acabou de ler?	GSV- Em uma escala de um a cinco, onde 1 é muito complexo e 5 é muito simples, como você classificaria a complexidade do texto que acabou de ler?	ST2 Em uma escala de um a cinco, onde 1 é muito complexo e 5 é muito simples, como você classificaria a complexidade do texto que acabou de ler?	RT2- Em uma escala de um a cinco, onde 1 é muito complexo e 5 é muito simples, como você classificaria a complexidade do texto que acabou de ler?	HCL- Em uma escala de um a cinco, onde 1 é muito complexo e 5 é muito simples, como você classificaria a complexidade do texto que acabou de ler?	QUI- Em uma escala de um a cinco, onde 1 é texto sem coerência nenhuma e 5 é texto com muita coerência, como você avalia a coerência do texto 1?	GSV- Em uma escala de um a cinco, onde 1 é texto sem coerência nenhuma e 5 é texto com muita coerência, como você avalia a coerência do texto 2?	ST2- Em uma escala de um a cinco, onde 1 é texto sem coerência nenhuma e 5 é texto com muita coerência, como você avalia a coerência do texto 3?	RT2- Em uma escala de um a cinco, onde 1 é texto sem coerência nenhuma e 5 é texto com muita coerência, como você avalia a coerência do texto 4?	HCL- Em uma escala de um a cinco, onde 1 é texto sem coerência nenhuma e 5 é texto com muita coerência, como você avalia a coerência do texto 5?
1002154383	12/11/2018	Feminino	22	São Paulo	São Paulo	Sudeste	Ensino Superi	3	1- Muito complexo.	1- Muito complexo.	1- Muito complexo.	3	3	2	1 - Texto sem coerência nenhuma	3	5 - Texto com muita coerência.
1003125879	09/11/2018	Masculino	20	Belém	Pará	Norte	Ensino Superi	5 - Muito simples.	2	5 - Muito simples.	2	5 - Muito simples.	5 - Texto com muita coerência.	3	5 - Texto com muita coerência.	1 - Texto sem coerência nenhuma	5 - Texto com muita coerência.
1004860692	12/11/2018	Masculino	36	João de M	io de Janei	Sudeste	Não Ensino Su	5 - Muito simples.	4	5 - Muito simples.	1- Muito complexo.	5 - Muito simples.	5 - Texto com muita coerência.	5 - Texto com muita coerência.	5 - Texto com muita coerência.	2	5 - Texto com muita coerência.
1004905168	09/11/2018	Masculino	25	Campinas	São Paulo	Sudeste	Ensino Superi	3	3	2	3	3	4	2	5 - Texto com muita coerência.	3	5 - Texto com muita coerência.
1005925071	12/11/2018	Feminino	38	Curitiba	Paraná	Sul	Ensino Superi	4	2	5 - Muito simples.	1- Muito complexo.	3	5 - Texto com muita coerência.	2	5 - Texto com muita coerência.	2	4
1006440182	13/11/2018	Feminino	36	Campinas	São Paulo	Sudeste	Ensino Superi	3	3	3	1- Muito complexo.	3	4	3	4	1 - Texto sem coerência nenhuma	4
1006831504	09/11/2018	Feminino	36	Sumaré	São Paulo	Sudeste	Não Ensino Su	3	3	2	3	2	3	3	2	2	2
1006857691	10/11/2018	Feminino	40	Salvador	Bahia	Nordeste	Ensino Superi	3	2	4	2	2	5 - Texto com muita coerência.	3	4	1 - Texto sem coerência nenhuma	4
1008829997	12/11/2018	Masculino	51	Guarabira	Paraíba	Nordeste	Não Ensino Su	3	3	4	3	3	3	3	3	3	4
1012445443	09/11/2018	Feminino	24	Parauapeba	Pará	Norte	Não Ensino Su	3	2	4	2	2	4	3	4	2	4
1012715833	11/11/2018	Masculino	23	zeiro do Oe	Paraná	Sul	Ensino Superi	4	4	3	3	4	3	5 - Texto com muita coerência.	4	3	3
1019220710	09/11/2018	Feminino	46	Guarulhos	São Paulo	Sudeste	Ensino Superi	4	1- Muito complexo.	4	1- Muito complexo.	3	2	3	4	5 - Texto com muita coerência.	2
1020219802	10/11/2018	Feminino	54	Montes Clar	Minas Gerai	Sudeste	Ensino Superi	5 - Muito simples.	2	5 - Muito simples.	2	4	5 - Texto com muita coerência.	4	5 - Texto com muita coerência.	1 - Texto sem coerência nenhuma	5 - Texto com muita coerência.
1020297008	09/11/2018	Masculino	27	Ivoti	Grande do	Sul	Ensino Superi	4	4	3	3	4	3	5 - Texto com muita coerência.	3	1 - Texto sem coerência nenhuma	5 - Texto com muita coerência.
1020509443	09/11/2018	Feminino	25	Fortaleza	Ceará	Nordeste	Não Ensino Su	5 - Muito simples.	1- Muito complexo.	5 - Muito simples.	1- Muito complexo.	3	5 - Texto com muita coerência.	1 - Texto sem coerência nenhuma	5 - Texto com muita coerência.	1 - Texto sem coerência nenhuma	4
1020972385	12/11/2018	Feminino	48	pucaia do S	Grande do	Sul	Não Ensino Su	5 - Muito simples.	1- Muito complexo.	1- Muito complexo.	1- Muito complexo.	5 - Muito simples.	5 - Texto com muita coerência.	1 - Texto sem coerência nenhuma	5 - Texto com muita coerência.	1 - Texto sem coerência nenhuma	5 - Texto com muita coerência.
1023867602	13/11/2018	Feminino	46	Viçosa	Minas Gerai	Sudeste	Não Ensino Su	5 - Muito simples.	1- Muito complexo.	5 - Muito simples.	1- Muito complexo.	5 - Muito simples.	4	3	5 - Texto com muita coerência.	5 - Texto com muita coerência.	5 - Texto com muita coerência.
1025578129	12/11/2018	Feminino	19	araguá do S	anta Catari	Sul	Ensino Superi	3	2	4	2	4	3	3	3	3	3
1026706924	12/11/2018	Feminino	45	Atibaia	São Paulo	Sudeste	Não Ensino Su	3	2	5 - Muito simples.	1- Muito complexo.	3	5 - Texto com muita coerência.	3	5 - Texto com muita coerência.	1 - Texto sem coerência nenhuma	4
1030130958	12/11/2018	Feminino	49	io de Janei	io de Janei	Sudeste	Não Ensino Su	3	2	5 - Muito simples.	1- Muito complexo.	3	2	4	1 - Texto sem coerência nenhuma	5 - Texto com muita coerência.	3
1032199778	12/11/2018	Feminino	29	Jequié	Bahia	Nordeste	Não Ensino Su	2	1- Muito complexo.	2	1- Muito complexo.	2	4	2	4	1 - Texto sem coerência nenhuma	4
1035086301	11/11/2018	Feminino	46	Guarulhos	São Paulo	Sudeste	Ensino Superi	4	1- Muito complexo.	5 - Muito simples.	1- Muito complexo.	3	5 - Texto com muita coerência.	1 - Texto sem coerência nenhuma	5 - Texto com muita coerência.	1 - Texto sem coerência nenhuma	3
1035097097	09/11/2018	Feminino	28	Lages	anta Catari	Sul	Não Ensino Su	1- Muito complexo.	1- Muito complexo.	1- Muito complexo.	1- Muito complexo.	1- Muito complexo.	5 - Texto com muita coerência.	5 - Texto com muita coerência.	5 - Texto com muita coerência.	5 - Texto com muita coerência.	5 - Texto com muita coerência.
1036269399	09/11/2018	Masculino	29	Olinda	Pernambuc	Nordeste	Ensino Superi	3	3	3	3	3	3	3	3	3	3
1036709025	09/11/2018	Feminino	42	io de Janei	io de Janei	Sudeste	Ensino Superi	3	2	1- Muito complexo.	2	2	3	4	4	4	4
1037490250	11/11/2018	Masculino	36	Carapicuíba	São Paulo	Sudeste	Não Ensino Su	2	2	4	3	4	4	2	5 - Texto com muita coerência.	2	1 - Texto sem coerência nenhuma
1037757798	09/11/2018	Masculino	37	São Paulo	São Paulo	Sudeste	Não Ensino Su	1- Muito complexo.	1- Muito complexo.	5 - Muito simples.	2	5 - Muito simples.	1 - Texto sem coerência nenhuma	1 - Texto sem coerência nenhuma	1 - Texto sem coerência nenhuma	1 - Texto sem coerência nenhuma	1 - Texto sem coerência nenhuma
1037774244	09/11/2018	Masculino	44	Curitiba	Paraná	Sul	Não Ensino Su	4	1- Muito complexo.	4	1- Muito complexo.	1- Muito complexo.	1 - Texto sem coerência nenhuma	5 - Texto com muita coerência.	2	4	2
1039265817	09/11/2018	Masculino	37	chão das Ne	Bahia	Nordeste	Não Ensino Su	3	4	4	3	3	3	4	4	4	3
1040582098	12/11/2018	Feminino	18	Gravataí	Grande do	Sul	Não Ensino Su	1- Muito complexo.	1- Muito complexo.	1- Muito complexo.	1- Muito complexo.	1- Muito complexo.	1 - Texto sem coerência nenhuma	4	1 - Texto sem coerência nenhuma	1 - Texto sem coerência nenhuma	3
1041640338	09/11/2018	Masculino	48	Santo Andr	São Paulo	Sudeste	Ensino Superi	3	2	4	1- Muito complexo.	3	5 - Texto com muita coerência.	4	5 - Texto com muita coerência.	2	5 - Texto com muita coerência.
1042629377	09/11/2018	Masculino	46	Fortaleza	Ceará	Nordeste	Ensino Superi	1- Muito complexo.	2	2	1- Muito complexo.	3	5 - Texto com muita coerência.	1 - Texto sem coerência nenhuma	5 - Texto com muita coerência.	2	4
1044097433	09/11/2018	Feminino	37	Salvador	Bahia	Nordeste	Ensino Superi	1- Muito complexo.	1- Muito complexo.	2	1- Muito complexo.	3	3	3	5 - Texto com muita coerência.	3	5 - Texto com muita coerência.
1044663133	12/11/2018	Feminino	33	Jequié	Bahia	Nordeste	Não Ensino Su	4	5 - Muito simples.	4	2	5 - Muito simples.	4	2	1 - Texto sem coerência nenhuma	4	1 - Texto sem coerência nenhuma
1046413546	09/11/2018	Feminino	19	ão Leopold	Grande do	Sul	Não Ensino Su	5 - Muito simples.	2	5 - Muito simples.	3	3	5 - Texto com muita coerência.	3	4	3	3
1048743588	09/11/2018	Masculino	18	Junqueiro	Alagoas	Nordeste	Não Ensino Su	5 - Muito simples.	4	5 - Muito simples.	4	5 - Muito simples.	5 - Texto com muita coerência.	1 - Texto sem coerência nenhuma	5 - Texto com muita coerência.	1 - Texto sem coerência nenhuma	5 - Texto com muita coerência.
1051267890	09/11/2018	Feminino	33	Araras	São Paulo	Sudeste	Ensino Superi	1- Muito complexo.	1- Muito complexo.	3	3	3	3	1 - Texto sem coerência nenhuma	4	2	3
1055919518	12/11/2018	Masculino	18	Goiânia	Goiás	Centro Oest	Não Ensino Su	3	2	3	4	3	4	2	5 - Texto com muita coerência.	1 - Texto sem coerência nenhuma	4
1056270696	10/11/2018	Feminino	19	Praia Grand	São Paulo	Sudeste	Não Ensino Su	5 - Muito simples.	2	5 - Muito simples.	5 - Muito simples.	5 - Muito simples.	5 - Texto com muita coerência.	5 - Texto com muita coerência.	5 - Texto com muita coerência.	5 - Texto com muita coerência.	5 - Texto com muita coerência.
1059737571	09/11/2018	Feminino	18	Guamiranga	Paraná	Sul	Não Ensino Su	2	3	3	3	3	3	2	3	3	2
1061729700	12/11/2018	Feminino	42	São Paulo	São Paulo	Sudeste	Ensino Superi	4	2	3	1- Muito complexo.	4	3	1 - Texto sem coerência nenhuma	4	1 - Texto sem coerência nenhuma	1 - Texto sem coerência nenhuma
1062301297	10/11/2018	Feminino	39	Helena de	Goiás	Centro Oest	Não Ensino Su	1- Muito complexo.	3	1- Muito complexo.	3	1- Muito complexo.	5 - Texto com muita coerência.	3	5 - Texto com muita coerência.	3	5 - Texto com muita coerência.
1063010767	12/11/2018	Masculino	52	Mesquita	io de Janei	Sudeste	Não Ensino Su	3	3	3	3	3	1 - Texto sem coerência nenhuma	3	4	2	4
1065399637	12/11/2018	Masculino	29	nçalo do Ar	Grande do N	Nordeste	Não Ensino Su	4	1- Muito complexo.	3	1- Muito complexo.	2	4	4	4	2	3
1068427722	12/11/2018	Feminino	19	Goianésia	Goiás	Centro Oest	Não Ensino Su	5 - Muito simples.	5 - Muito simples.	5 - Muito simples.	1- Muito complexo.	4	5 - Texto com muita coerência.	3	5 - Texto com muita coerência.	1 - Texto sem coerência nenhuma	5 - Texto com muita coerência.
1068469694	09/11/2018	Masculino	22	mpina Gran	Paraíba	Nordeste	Não Ensino Su	4	4	3	3	3	3	4	4	4	4
1071014788	09/11/2018	Feminino	31	São Paulo	São Paulo	Sudeste	Ensino Superi	4	2	5 - Muito simples.	1- Muito complexo.	5 - Muito simples.	5 - Texto com muita coerência.	3	5 - Texto com muita coerência.	2	5 - Texto com muita coerência.
1071181318	12/11/2018	Masculino	55	Petrópolis	io de Janei	Sudeste	Não Ensino Su	5 - Muito simples.	3	5 - Muito simples.	1- Muito complexo.	4	4	1 - Texto sem coerência nenhuma	5 - Texto com muita coerência.	1 - Texto sem coerência nenhuma	4
1071909432	12/11/2018	Masculino	23	Laguna	anta Catari	Sul	Não Ensino Su	2	5 - Muito simples.	5 - Muito simples.	1- Muito complexo.	4	3	3	4	5 - Texto com muita coerência.	2
1075351294	12/11/2018	Feminino	51	São Paulo	São Paulo	Sudeste	Não Ensino Su	5 - Muito simples.	5 - Muito simples.	1- Muito complexo.	5 - Muito simples.	5 - Muito simples.	5 - Texto com muita coerência.	1 - Texto sem coerência nenhuma	1 - Texto sem coerência nenhuma	1 - Texto sem coerência nenhuma	1 - Texto sem coerência nenhuma
1076431146	10/11/2018	Masculino	36	São Paulo	São Paulo	Sudeste	Ensino Superi	3	2	3	4	2	2	3	2	4	4
1077249618	09/11/2018	Masculino	41	São Gonçalo	io de Janei	Sudeste	Não Ensino Su	3	2	5 - Muito simples.	1- Muito complexo.	5 - Muito simples.	3	2	2	4	1 - Texto sem coerência nenhuma
1082329715	13/11/2018	Masculino	32	Botucatu	São Paulo	Sudeste	Não Ensino Su	3	5 - Muito simples.	3	5 - Muito simples.	4	1 - Texto sem coerência nenhuma	2	4	2	3
1084338654	09/11/2018	Masculino	18	São José	anta Catari	Sul	Não Ensino Su	1- Muito complexo.	1- Muito complexo.	5 - Muito simples.	1- Muito complexo.	1- Muito complexo.	3	5 - Texto com muita coerência.	5 - Texto com muita coerência.	3	3
1086321196	12/11/2018	Feminino	44	Bariri	São Paulo	Sudeste	Não Ensino Su	2	4	4	1- Muito complexo.	1- Muito complexo.	3	3	1 - Texto sem coerência nenhuma	5 - Texto com muita coerência.	4
1086977576	12/11/2018	Masculino	42	Salvador	Bahia	Nordeste	Ensino Superi	1- Muito complexo.	1- Muito complexo.	5 - Muito simples.	5 - Muito simples.	5 - Muito simples.	5 - Texto com muita coerência.	5 - Texto com muita coerência.	1 - Texto sem coerência nenhuma	2	1 - Texto sem coerência nenhuma
1087976213	12/11/2018	Masculino	33	árzea Gran	Mato Gross	Centro Oest	Não Ensino Su	3	3	3	3	3	3	3	3	3	3
1088679089	09/11/2018	Masculino	54	Fortaleza	Ceará	Nordeste	Ensino Superi	1- Muito complexo.	5 - Muito simples.	5 - Muito simples.	1- Muito complexo.	1- Muito complexo.	5 - Texto com muita coerência.	5 - Texto com muita coerência.	5 - Texto com muita coerência.	5 - Texto com muita coerência.	5 - Texto com muita coerência.
1089785551	12/11/2018	Feminino	22	Campinas	São Paulo	Sudeste	Não Ensino Su	4	3	5 - Muito simples.	1- Muito complexo.	4	3	2	4	2	3
1090250928	10/11/2018	Masculino	41	Campinas	São Paulo	Sudeste	Não Ensino Su	3	5 - Muito simples.	5 - Muito simples.	3	1- Muito complexo.	4	1 - Texto sem coerência nenhuma	5 - Texto com muita coerência.	1 - Texto sem coerência nenhuma	4
1091044899	11/11/2018	Masculino	39	Porto Alegr	Grande do	Sul	Ensino Superi	2	1- Muito complexo.	3	1- Muito complexo.	1- Muito complexo.	3	3	3	1 - Texto sem coerência nenhuma	3
1091284449	12/11/2018	Feminino	31	Natal	Grande do N	Nordeste	Não Ensino Su	2	2	1- Muito complexo.	1- Muito complexo.	1- Muito complexo.	5 - Texto com muita coerência.	1 - Texto sem coerência nenhuma	3	3	4
1095454295	12/11/2018	Feminino	21	Guará	strito Fede	Centro Oest	Ensino Superi	1- Muito complexo.	3	5 - Muito simples.	4	4	5 - Texto com muita coerência.	5 - Texto com muita coerência.	5 - Texto com muita coerência.	3	4
1098500237	13/11/2018	Feminino	22	mpina Gran	Paraíba	Nordeste	Ensino Superi	3	3	5 - Muito simples.	1- Muito complexo.	5 - Muito simples.	5 - Texto com muita coerência.	2	5 - Texto com muita coerência.	1 - Texto sem coerência nenhuma	4
1099179583	12/11/2018	Feminino	34	Curitiba	Paraná	Sul	Não Ensino Su	3	3	3	2	3	3	3	3	3	4
1103112519	09/11/2018	Feminino	40	Salvador	Bahia	Nordeste	Não Ensino Su	1- Muito complexo.	1- Muito complexo.	1- Muito complexo.	1- Muito complexo.	1- Muito complexo.	5 - Texto com muita coerência.	5 - Texto com muita coerência.	5 - Texto com muita coerência.	5 - Texto com muita coerência.	5 - Texto com muita coerência.
1104331303	09/11/2018	Masculino	22	Diadema	São Paulo	Sudeste	Ensino Superi	5 - Muito simples.	4	5 - Muito simples.	2	4	3	2	5 - Texto com muita coerência.	1 - Texto sem coerência nenhuma	4
1105609031	10/11/2018	Masculino	51	Arapiraca	Alagoas	Nordeste	Ensino Superi	4	3	5 - Muito simples.	5 - Muito simples.	3	1 - Texto sem coerência nenhuma	4	1 - Texto sem coerência nenhuma	1 - Texto sem coerência nenhuma	1 - Texto sem coerência nenhuma
1105759627	11/11/2018	Feminino	34	ampo Gran	o Grosso d	Centro Oest	Não Ensino Su	4	1- Muito complexo.	3	2	1- Muito complexo.	3	3	3	3	3
1106119731	10/11/2018	Feminino	21	Botucatu	São Paulo	Sudeste	Não Ensino Su	2	2	4	1- Muito complexo.	1- Muito complexo.	5 - Texto com muita coerência.	1 - Texto sem coerência nenhuma	4	1 - Texto sem coerência nenhuma	4
1109622782	13/11/2018	Feminino	25	Vilhena	Rondônia	Norte	Ensino Superi	1- Muito complexo.	1- Muito complexo.	2	3	2	2	1 - Texto sem coerência nenhuma	5 - Texto com muita coerência.	2	4
1111888627	12/11/2018	Masculino	36	São Paulo	São Paulo	Sudeste	Não Ensino Su	2	3	2	2	5 - Muito simples.	3	4	4	3	4
1113988866	09/11/2018	Feminino	32	Patrocínio	Minas Gerai	Sudeste	Não Ensino Su	5 - Muito simples.	5 - Muito simples.	1- Muito complexo.	5 - Muito simples.	1- Muito complexo.	5 - Texto com muita coerência.	1 - Texto sem coerência nenhuma	5 - Texto com muita coerência.	5 - Texto com muita coerência.	1 - Texto sem coerência nenhuma
1114303351	09/11/2018	Masculino	38	io de Janei	io de Janei	Sudeste	Ensino Superi	4	1- Muito complexo.	4	1- Muito complexo.	4	4	3	5 - Texto com muita coerência.	1 - Texto sem coerência nenhuma	5 - Texto com muita coerência.
1115293054	11/11/2018	Feminino	27	árzea Gran	Mato Gross	Centro Oest	Ensino Superi	3	1- Muito complexo.	4	1- Muito complexo.	2	5 - Texto com muita coerência.	2	5 - Texto com muita coerência.	2	4
1115563782	09/11/2018	Masculino	27	Catalão	Goiás	Centro Oest	Não Ensino Su	3	3	1- Muito complexo.	3	2	4	4	4	4	4
1116594921	10/11/2018	Feminino	45	Igarassu	Pernambuc	Nordeste	Não Ensino Su	3	2	4	1- Muito complexo.	2	5 - Texto com muita coerência.	4	5 - Texto com muita coerência.	1 - Texto sem coerência nenhuma	4

1116800233	09/11/2018	Feminino	37	ampo Grand	o Grosso d	entro Oest	Ensino Superi	4	3	5 - Muito simples.	1- Muito complexo.	3	4	2	4	1 - Texto sem coerência nenhuma	4
1117056051	10/11/2018	Masculino	26	Salgueiro	Pernambuc	Nordeste	Não Ensino Su	1- Muito complexo.	4	2	1- Muito complexo.	3	5 - Texto com muita coerência.	2	5 - Texto com muita coerência.	2	3
1117874744	09/11/2018	Feminino	32	Osasco	São Paulo	Sudeste	Ensino Superi	3	2	3	3	3	3	3	3	3	3
1122719585	09/11/2018	Masculino	32	Belém	Pará	Norte	Não Ensino Su	4	1- Muito complexo.	3	1- Muito complexo.	1- Muito complexo.	4	4	5 - Texto com muita coerência.	4	4
1123223067	12/11/2018	Feminino	20	Jacareí	São Paulo	Sudeste	Não Ensino Su	1- Muito complexo.	1- Muito complexo.	1- Muito complexo.	1- Muito complexo.	1- Muito complexo.	1 - Texto sem coerência nenhuma	1 - Texto sem coerência nenhuma	5 - Texto com muita coerência.	1 - Texto sem coerência nenhuma	1 - Texto sem coerência nenhuma
1123568654	09/11/2018	Masculino	32	Florianópoli	anta Catarin	Sul	Ensino Superi	4	2	4	1- Muito complexo.	5 - Muito simples.	4	3	5 - Texto com muita coerência.	1 - Texto sem coerência nenhuma	5 - Texto com muita coerência.
1125144495	12/11/2018	Masculino	27	Curitiba	Paraná	Sul	Ensino Superi	3	3	3	3	2	4	3	2	2	2
1128186911	09/11/2018	Feminino	40	Manaus	Amazonas	Norte	Ensino Superi	3	3	3	3	3	5 - Texto com muita coerência.	4	5 - Texto com muita coerência.	3	5 - Texto com muita coerência.
1128371430	09/11/2018	Masculino	37	Osasco	São Paulo	Sudeste	Ensino Superi	4	3	4	1- Muito complexo.	2	2	1 - Texto sem coerência nenhuma	4	1 - Texto sem coerência nenhuma	1 - Texto sem coerência nenhuma
1128784788	12/11/2018	Masculino	42	Macaé	io de Janeir	Sudeste	Não Ensino Su	2	2	2	4	1- Muito complexo.	3	3	4	5 - Texto com muita coerência.	1 - Texto sem coerência nenhuma
1129052490	12/11/2018	Masculino	39	Contagem	Minas Gerai	Sudeste	Ensino Superi	3	1- Muito complexo.	5 - Muito simples.	1- Muito complexo.	4	4	1 - Texto sem coerência nenhuma	5 - Texto com muita coerência.	1 - Texto sem coerência nenhuma	3
1131089722	09/11/2018	Feminino	27	Nova Iguaçu	io de Janeir	Sudeste	Não Ensino Su	4	3	5 - Muito simples.	1- Muito complexo.	4	5 - Texto com muita coerência.	4	4	2	4
1132356309	11/11/2018	Masculino	41	io de Janeir	io de Janeir	Sudeste	Não Ensino Su	5 - Muito simples.	5 - Muito simples.	5 - Muito simples.	5 - Muito simples.	5 - Muito simples.	5 - Texto com muita coerência.	5 - Texto com muita coerência.	5 - Texto com muita coerência.	5 - Texto com muita coerência.	5 - Texto com muita coerência.
1134118936	10/11/2018	Feminino	31	Colombo	Paraná	Sul	Ensino Superi	4	3	3	2	3	3	2	3	3	2
1135906876	09/11/2018	Masculino	38	Aparecida	São Paulo	Sudeste	Ensino Superi	3	3	3	3	3	3	3	3	3	3
1136496417	12/11/2018	Feminino	28	Muriaé	Minas Gerai	Sudeste	Não Ensino Su	4	3	5 - Muito simples.	1- Muito complexo.	4	5 - Texto com muita coerência.	4	5 - Texto com muita coerência.	5 - Texto com muita coerência.	5 - Texto com muita coerência.
1137095087	09/11/2018	Feminino	18	Aliança	Pernambuc	Nordeste	Não Ensino Su	3	4	1- Muito complexo.	1- Muito complexo.	5 - Muito simples.	5 - Texto com muita coerência.	1 - Texto sem coerência nenhuma	1 - Texto sem coerência nenhuma	2	2
1145818089	09/11/2018	Feminino	23	João Câmar	Grande do N	Nordeste	Não Ensino Su	5 - Muito simples.	2	5 - Muito simples.	2	4	5 - Texto com muita coerência.	3	3	1 - Texto sem coerência nenhuma	5 - Texto com muita coerência.
1146273997	09/11/2018	Feminino	44	São Paulo	São Paulo	Sudeste	Não Ensino Su	3	2	2	1- Muito complexo.	2	2	2	1 - Texto sem coerência nenhuma	1 - Texto sem coerência nenhuma	1 - Texto sem coerência nenhuma
1146528960	09/11/2018	Feminino	20	João Pessoa	Paraíba	Nordeste	Não Ensino Su	4	4	5 - Muito simples.	2	4	5 - Texto com muita coerência.	5 - Texto com muita coerência.	5 - Texto com muita coerência.	3	5 - Texto com muita coerência.
1149693211	12/11/2018	Masculino	54	mpos do Jor	São Paulo	Sudeste	Não Ensino Su	4	3	4	4	4	4	3	4	4	4
1149941316	09/11/2018	Masculino	27	Sumaré	São Paulo	Sudeste	Ensino Superi	4	2	5 - Muito simples.	5 - Muito simples.	2	5 - Texto com muita coerência.	5 - Texto com muita coerência.	5 - Texto com muita coerência.	1 - Texto sem coerência nenhuma	5 - Texto com muita coerência.
1151506399	11/11/2018	Masculino	22	São José	anta Catarin	Sul	Ensino Superi	5 - Muito simples.	2	4	4	4	5 - Texto com muita coerência.	3	5 - Texto com muita coerência.	2	1 - Texto sem coerência nenhuma
1153156646	09/11/2018	Feminino	39	São Paulo	São Paulo	Sudeste	Ensino Superi	4	3	3	1- Muito complexo.	3	5 - Texto com muita coerência.	4	5 - Texto com muita coerência.	1 - Texto sem coerência nenhuma	4
1154164979	09/11/2018	Feminino	43	São Paulo	São Paulo	Sudeste	Ensino Superi	3	1- Muito complexo.	4	1- Muito complexo.	2	5 - Texto com muita coerência.	3	5 - Texto com muita coerência.	1 - Texto sem coerência nenhuma	5 - Texto com muita coerência.
1155485035	10/11/2018	Feminino	21	Salvador	Bahia	Nordeste	Ensino Superi	4	3	5 - Muito simples.	2	4	5 - Texto com muita coerência.	4	4	2	5 - Texto com muita coerência.
1156859188	12/11/2018	Feminino	37	io de Janeir	io de Janeir	Sudeste	Ensino Superi	5 - Muito simples.	2	5 - Muito simples.	1- Muito complexo.	3	4	1 - Texto sem coerência nenhuma	5 - Texto com muita coerência.	2	4
1157214043	12/11/2018	Feminino	47	io de Janeir	io de Janeir	Sudeste	Não Ensino Su	3	2	3	2	3	5 - Texto com muita coerência.	5 - Texto com muita coerência.	5 - Texto com muita coerência.	1 - Texto sem coerência nenhuma	5 - Texto com muita coerência.
1157698518	13/11/2018	Feminino	51	Escada	Pernambuc	Nordeste	Não Ensino Su	5 - Muito simples.	5 - Muito simples.	5 - Muito simples.	5 - Muito simples.	5 - Muito simples.	1 - Texto sem coerência nenhuma	5 - Texto com muita coerência.	5 - Texto com muita coerência.	5 - Texto com muita coerência.	5 - Texto com muita coerência.
1158756026	12/11/2018	Masculino	20	io de Janeir	io de Janeir	Sudeste	Não Ensino Su	2	2	3	2	3	3	3	4	3	4
1159079813	10/11/2018	Feminino	25	io de Janeir	io de Janeir	Sudeste	Ensino Superi	5 - Muito simples.	1- Muito complexo.	4	1- Muito complexo.	4	5 - Texto com muita coerência.	2	5 - Texto com muita coerência.	1 - Texto sem coerência nenhuma	4
1159592905	10/11/2018	Masculino	36	o do Rodrig	Grande do N	Nordeste	Ensino Superi	4	4	3	4	4	4	4	3	4	4
1159766584	09/11/2018	Masculino	36	Mirassolând	São Paulo	Sudeste	Não Ensino Su	3	3	3	3	3	3	3	3	3	3
1159818830	09/11/2018	Feminino	21	Brasília	strito Fede	entro Oest	Ensino Superi	3	4	5 - Muito simples.	1- Muito complexo.	1- Muito complexo.	5 - Texto com muita coerência.	3	5 - Texto com muita coerência.	2	5 - Texto com muita coerência.
1163155599	12/11/2018	Feminino	23	Porto Alegr	Grande do	Sul	Não Ensino Su	4	1- Muito complexo.	3	1- Muito complexo.	3	5 - Texto com muita coerência.	1 - Texto sem coerência nenhuma	5 - Texto com muita coerência.	3	4
1165106218	09/11/2018	Feminino	35	Manaus	Amazonas	Norte	Não Ensino Su	5 - Muito simples.	2	5 - Muito simples.	1- Muito complexo.	5 - Muito simples.	5 - Texto com muita coerência.	3	5 - Texto com muita coerência.	1 - Texto sem coerência nenhuma	5 - Texto com muita coerência.
1165199726	12/11/2018	Masculino	25	Joinville	anta Catarin	Sul	Não Ensino Su	3	2	4	1- Muito complexo.	4	3	3	5 - Texto com muita coerência.	2	2
1165391985	09/11/2018	Feminino	35	ira de Santa	Bahia	Nordeste	Não Ensino Su	2	1- Muito complexo.	3	5 - Muito simples.	1- Muito complexo.	3	4	2	1 - Texto sem coerência nenhuma	5 - Texto com muita coerência.
1165586493	10/11/2018	Feminino	25	Sumaré	São Paulo	Sudeste	Ensino Superi	1- Muito complexo.	3	5 - Muito simples.	2	5 - Muito simples.	5 - Texto com muita coerência.	3	5 - Texto com muita coerência.	3	4
1166167444	12/11/2018	Feminino	23	Giruá	Grande do	Sul	Não Ensino Su	1- Muito complexo.	1- Muito complexo.	5 - Muito simples.	1- Muito complexo.	1- Muito complexo.	3	2	5 - Texto com muita coerência.	3	4
1168357411	10/11/2018	Masculino	38	io de Janeir	io de Janeir	Sudeste	Ensino Superi	4	2	4	2	3	5 - Texto com muita coerência.	3	5 - Texto com muita coerência.	4	4
1169353981	09/11/2018	Feminino	36	Ponta Gross	Paraná	Sul	Não Ensino Su	4	3	5 - Muito simples.	1- Muito complexo.	4	5 - Texto com muita coerência.	4	4	1 - Texto sem coerência nenhuma	2
1169594340	10/11/2018	Feminino	35	Jandira	São Paulo	Sudeste	Ensino Superi	3	1- Muito complexo.	4	1- Muito complexo.	3	3	3	4	1 - Texto sem coerência nenhuma	5 - Texto com muita coerência.
1169786337	09/11/2018	Feminino	19	Antônio de	Bahia	Nordeste	Não Ensino Su	3	3	4	1- Muito complexo.	3	5 - Texto com muita coerência.	3	5 - Texto com muita coerência.	1 - Texto sem coerência nenhuma	5 - Texto com muita coerência.
1172217587	12/11/2018	Masculino	21	Eirunepé	Amazonas	Norte	Não Ensino Su	4	2	4	1- Muito complexo.	4	5 - Texto com muita coerência.	4	5 - Texto com muita coerência.	1 - Texto sem coerência nenhuma	5 - Texto com muita coerência.
1173970435	09/11/2018	Feminino	31	Cotia	São Paulo	Sudeste	Ensino Superi	3	1- Muito complexo.	2	1- Muito complexo.	3	4	4	5 - Texto com muita coerência.	1 - Texto sem coerência nenhuma	5 - Texto com muita coerência.
1174082288	10/11/2018	Feminino	57	São Paulo	São Paulo	Sudeste	Não Ensino Su	3	3	1- Muito complexo.	3	3	3	3	3	1 - Texto sem coerência nenhuma	4
1174681476	09/11/2018	Feminino	39	Brasília	strito Fede	entro Oest	Ensino Superi	3	2	4	1- Muito complexo.	3	4	2	5 - Texto com muita coerência.	1 - Texto sem coerência nenhuma	5 - Texto com muita coerência.
1174782763	12/11/2018	Feminino	24	São Paulo	São Paulo	Sudeste	Ensino Superi	3	2	4	3	4	4	3	3	2	4
1174811395	09/11/2018	Feminino	36	io de Janeir	io de Janeir	Sudeste	Ensino Superi	3	3	3	2	3	3	3	5 - Texto com muita coerência.	4	3
1177366381	11/11/2018	Masculino	22	Campinas	São Paulo	Sudeste	Não Ensino Su	3	3	3	3	3	3	3	3	3	3
1177970353	09/11/2018	Masculino	20	Jequié	Bahia	Nordeste	Não Ensino Su	1- Muito complexo.	1- Muito complexo.	3	1- Muito complexo.	3	2	2	3	2	4
1179233744	13/11/2018	Feminino	52	osé do Rio	São Paulo	Sudeste	Não Ensino Su	3	3	5 - Muito simples.	1- Muito complexo.	3	5 - Texto com muita coerência.	3	1 - Texto sem coerência nenhuma	1 - Texto sem coerência nenhuma	3
1183161253	10/11/2018	Feminino	47	Salvador	Bahia	Nordeste	Não Ensino Su	4	1- Muito complexo.	3	1- Muito complexo.	5 - Muito simples.	5 - Texto com muita coerência.	5 - Texto com muita coerência.	5 - Texto com muita coerência.	1 - Texto sem coerência nenhuma	5 - Texto com muita coerência.
1183408513	10/11/2018	Masculino	43	Caxias	Maranhão	Nordeste	Ensino Superi	2	2	3	3	2	1 - Texto sem coerência nenhuma	2	2	2	2
1184366544	09/11/2018	Masculino	31	osé dos Ca	São Paulo	Sudeste	Não Ensino Su	3	3	5 - Muito simples.	3	3	3	2	4	2	3
1185048166	09/11/2018	Feminino	25	Brasília	strito Fede	entro Oest	Ensino Superi	2	1- Muito complexo.	4	1- Muito complexo.	3	5 - Texto com muita coerência.	3	4	2	4
1185136750	12/11/2018	Masculino	43	São Paulo	São Paulo	Sudeste	Não Ensino Su	1- Muito complexo.	1- Muito complexo.	5 - Muito simples.	1- Muito complexo.	5 - Muito simples.	5 - Texto com muita coerência.	5 - Texto com muita coerência.	3	3	3
1186298923	09/11/2018	Feminino	20	João Pessoa	Paraíba	Nordeste	Ensino Superi	5 - Muito simples.	2	5 - Muito simples.	1- Muito complexo.	3	4	4	4	1 - Texto sem coerência nenhuma	5 - Texto com muita coerência.
1188545002	12/11/2018	Feminino	18	Maranguap	Ceará	Nordeste	Não Ensino Su	3	2	4	4	2	2	4	2	3	2
1188856306	09/11/2018	Masculino	21	Feliz	Grande do	Sul	Ensino Superi	4	4	5 - Muito simples.	3	3	5 - Texto com muita coerência.	3	5 - Texto com muita coerência.	3	5 - Texto com muita coerência.
1189174629	09/11/2018	Feminino	18	Goiânia	Goiás	entro Oest	Não Ensino Su	3	1- Muito complexo.	5 - Muito simples.	1- Muito complexo.	3	4	4	5 - Texto com muita coerência.	2	4
1193881941	10/11/2018	Feminino	49	São Paulo	São Paulo	Sudeste	Ensino Superi	3	2	4	3	4	4	3	4	3	4
1194873543	09/11/2018	Feminino	33	Viamão	Grande do	Sul	Ensino Superi	5 - Muito simples.	3	5 - Muito simples.	1- Muito complexo.	4	5 - Texto com muita coerência.	3	5 - Texto com muita coerência.	1 - Texto sem coerência nenhuma	4
1195374800	09/11/2018	Feminino	43	São Paulo	São Paulo	Sudeste	Ensino Superi	5 - Muito simples.	1- Muito complexo.	5 - Muito simples.	1- Muito complexo.	5 - Muito simples.	4	1 - Texto sem coerência nenhuma	5 - Texto com muita coerência.	1 - Texto sem coerência nenhuma	5 - Texto com muita coerência.
1196339586	11/11/2018	Masculino	52	io de Janeir	io de Janeir	Sudeste	Não Ensino Su	3	3	3	2	3	4	4	4	1 - Texto sem coerência nenhuma	4
1196417700	12/11/2018	Masculino	26	quaquecetu	São Paulo	Sudeste	Não Ensino Su	3	3	5 - Muito simples.	1- Muito complexo.	3	5 - Texto com muita coerência.	4	5 - Texto com muita coerência.	4	5 - Texto com muita coerência.
1197256415	09/11/2018	Feminino	27	Goiânia	Goiás	entro Oest	Não Ensino Su	3	3	3	3	3	4	4	4	4	4
1197960460	09/11/2018	Feminino	40	São Paulo	São Paulo	Sudeste	Ensino Superi	4	4	4	2	4	5 - Texto com muita coerência.	5 - Texto com muita coerência.	5 - Texto com muita coerência.	1 - Texto sem coerência nenhuma	5 - Texto com muita coerência.
1198820839	12/11/2018	Feminino	34	Ananindeua	Pará	Norte	Não Ensino Su	3	4	5 - Muito simples.	1- Muito complexo.	3	5 - Texto com muita coerência.	3	5 - Texto com muita coerência.	1 - Texto sem coerência nenhuma	5 - Texto com muita coerência.
1199755408	09/11/2018	Feminino	30	Brasília	strito Fede	entro Oest	Ensino Superi	4	1- Muito complexo.	1- Muito complexo.	1- Muito complexo.	5 - Muito simples.	5 - Texto com muita coerência.	2	5 - Texto com muita coerência.	1 - Texto sem coerência nenhuma	5 - Texto com muita coerência.
1203782891	10/11/2018	Masculino	18	idente Prud	São Paulo	Sudeste	Ensino Superi	3	2	3	1- Muito complexo.	2	4	3	5 - Texto com muita coerência.	2	4
1204603085	12/11/2018	Feminino	36	Campinas	São Paulo	Sudeste	Ensino Superi	5 - Muito simples.	3	5 - Muito simples.	1- Muito complexo.	3	5 - Texto com muita coerência.	4	5 - Texto com muita coerência.	5 - Texto com muita coerência.	5 - Texto com muita coerência.
1207259292	09/11/2018	Masculino	20	Teresina	Piauí	Nordeste	Ensino Superi	5 - Muito simples.	3	4	3	2	4	4	4	4	4
1207292273	09/11/2018	Masculino	41	Petrópolis	io de Janeir	Sudeste	Ensino Superi	5 - Muito simples.	3	5 - Muito simples.	1- Muito complexo.	4	5 - Texto com muita coerência.	5 - Texto com muita coerência.	5 - Texto com muita coerência.	1 - Texto sem coerência nenhuma	5 - Texto com muita coerência.
1207566373	09/11/2018	Masculino	56	olta Redond	io de Janeir	Sudeste	Ensino Superi	3	4	3	1- Muito complexo.	2	2	2	4	2	4
1210558670	09/11/2018	Feminino	46	acho Fundo	strito Fede	entro Oest	Ensino Superi	4	2	4	2	2	4	2	4	3	3
1211276350	09/11/2018	Feminino	46	Santo Andr	São Paulo	Sudeste	Ensino Superi	3	2	4	1- Muito complexo.	4	5 - Texto com muita coerência.	1 - Texto sem coerência nenhuma	5 - Texto com muita coerência.	1 - Texto sem coerência nenhuma	3
1212171574	10/11/2018	Feminino	43	Itatiba	São Paulo	Sudeste	Ensino Superi	4	2	4	1- Muito complexo.	4	5 - Texto com muita coerência.	4	5 - Texto com muita coerência.	2	4
1214000598	09/11/2018	Feminino	31	Guará	strito Fede	entro Oest	Ensino Superi	5 - Muito simples.	5 - Muito simples.	5 - Muito simples.	1- Muito complexo.	5 - Muito simples.	5 - Texto com muita coerência.	1 - Texto sem coerência nenhuma	5 - Texto com muita coerência.	1 - Texto sem coerência nenhuma	5 - Texto com muita coerência.
1215274201	09/11/2018	Masculino	18	José de Pira	Paraíba	Nordeste	Ensino Superi	1- Muito complexo.	1- Muito complexo.	1- Muito complexo.	1- Muito complexo.	1- Muito complexo.	3	2	1 - Texto sem coerência nenhuma	5 - Texto com muita coerência.	4
1217727293	09/11/2018	Masculino	21	Cuiabá	Mato Gross	entro Oest	Ensino Superi	4	3	5 - Muito simples.	2	3	5 - Texto com muita coerência.	3	5 - Texto com muita coerência.	2	5 - Texto com muita coerência.
1220660411	09/11/2018	Masculino	37	Candeias	Bahia	Nordeste	Ensino Superi	1- Muito complexo.	2	1- Muito complexo.	2	1- Muito complexo.	5 - Texto com muita coerência.	2	4	5 - Texto com muita coerência.	2
1221182903	12/11/2018	Feminino	34	Carapicuíba	São Paulo	Sudeste	Ensino Superi	5 - Muito simples.	1- Muito complexo.	5 - Muito simples.	1- Muito complexo.	5 - Muito simples.	3	2	3	1 - Texto sem coerência nenhuma	4
1221578248	09/11/2018	Feminino	36	Fortaleza	Ceará	Nordeste	Ensino Superi	5 - Muito simples.	4	5 - Muito simples.	2	4	5 - Texto com muita coerência.	3	5 - Texto com muita coerência.	4	5 - Texto com muita coerência.
1221953912	09/11/2018	Feminino	39	Porto Alegr	Grande do	Sul	Não Ensino Su	4	2	2	3	3	4	4	4	3	4
1222316933	12/11/2018	Masculino	38	Cotia	São Paulo	Sudeste	Não Ensino Su	3	3	3	4	4	3	3	3	3	3
1224426343	10/11/2018	Masculino	45	Salvador	Bahia	Nordeste	Ensino Superi	5 - Muito simples.	3	4	3	5 - Muito simples.	5 - Texto com muita coerência.	1 - Texto sem coerência nenhuma	5 - Texto com muita coerência.	1 - Texto sem coerência nenhuma	5 - Texto com muita coerência.
1227963557	13/11/2018	Feminino	40	São José	anta Catarin	Sul	Não Ensino Su	3	3	3	2	4	2	3	2	3	3

1228605028	12/11/2018	Feminino	63	Fortaleza	Ceará	Nordeste	Não Ensino Su	5 - Muito simples.	5 - Muito simples.	5 - Muito simples.	3	5 - Muito simples.	5 - Texto com muita coerência.	5 - Texto com muita coerência.	5 - Texto com muita coerência.	1 - Texto sem coerência nenhuma	5 - Texto com muita coerência.
1228649412	10/11/2018	Masculino	34	Jaguariúna	São Paulo	Sudeste	Não Ensino Su	3	2	2	3	4	3	3	3	3	3
1230585840	12/11/2018	Masculino	43	Campinas	São Paulo	Sudeste	Ensino Superi	3	2	4	3	4	5 - Texto com muita coerência.	4	3	2	4
1230975336	12/11/2018	Masculino	40	Fortaleza	Ceará	Nordeste	Ensino Superi	3	2	3	1- Muito complexo.	5 - Muito simples.	3	3	4	3	3
1233985717	09/11/2018	Feminino	39	io de Janeir	io de Janeir	Sudeste	Ensino Superi	4	3	4	4	4	5 - Texto com muita coerência.	2	4	3	3
1234241179	11/11/2018	Masculino	40	Aracaju	Sergipe	Nordeste	Ensino Superi	4	3	5 - Muito simples.	1- Muito complexo.	3	5 - Texto com muita coerência.	2	5 - Texto com muita coerência.	3	4
1235337713	12/11/2018	Masculino	37	bastião do	Minas Gerai	Sudeste	Não Ensino Su	2	2	2	4	2	5 - Texto com muita coerência.	5 - Texto com muita coerência.	5 - Texto com muita coerência.	1 - Texto sem coerência nenhuma	5 - Texto com muita coerência.
1236118255	12/11/2018	Masculino	38	io de Janeir	io de Janeir	Sudeste	Não Ensino Su	3	2	4	3	4	3	3	4	4	4
1236768064	10/11/2018	Feminino	38	Quirinópoli	Goiás	Centro Oest	Ensino Superi	2	2	1- Muito complexo.	2	1- Muito complexo.	1 - Texto sem coerência nenhuma	5 - Texto com muita coerência.	4	2	5 - Texto com muita coerência.
1241754452	12/11/2018	Masculino	17	Antônio de	São Paulo	Sudeste	Não Ensino Su	3	2	3	4	3	3	4	2	4	2
1242148279	09/11/2018	Feminino	53	io de Janeir	io de Janeir	Sudeste	Não Ensino Su	1- Muito complexo.	1- Muito complexo.	3	1- Muito complexo.	2	4	3	4	1 - Texto sem coerência nenhuma	5 - Texto com muita coerência.
1243748264	12/11/2018	Masculino	40	osé dos Ca	São Paulo	Sudeste	Não Ensino Su	4	4	5 - Muito simples.	4	3	3	4	4	4	3
1245652548	09/11/2018	Feminino	20	Brasília	strito Fede	Centro Oest	Ensino Superi	3	2	5 - Muito simples.	1- Muito complexo.	3	4	2	5 - Texto com muita coerência.	1 - Texto sem coerência nenhuma	3
1246100058	10/11/2018	Feminino	30	Juazeiro	Bahia	Nordeste	Não Ensino Su	3	1- Muito complexo.	5 - Muito simples.	1- Muito complexo.	4	3	1 - Texto sem coerência nenhuma	3	1 - Texto sem coerência nenhuma	2
1247183910	09/11/2018	Masculino	68	Contagem	Minas Gerai	Sudeste	Não Ensino Su	2	2	1- Muito complexo.	3	2	1 - Texto sem coerência nenhuma	3	2	3	2
1247733196	12/11/2018	Feminino	25	Blumenau	anta Catari	Sul	Não Ensino Su	3	1- Muito complexo.	5 - Muito simples.	4	4	3	3	3	3	3
1248784637	09/11/2018	Feminino	20	Natal	Grande do N	Nordeste	Ensino Superi	3	1- Muito complexo.	3	1- Muito complexo.	1- Muito complexo.	5 - Texto com muita coerência.	2	5 - Texto com muita coerência.	3	2
1250003404	10/11/2018	Masculino	54	Sete Lagoas	Minas Gerai	Sudeste	Ensino Superi	4	3	4	4	3	2	3	5 - Texto com muita coerência.	4	4
1251295560	10/11/2018	Feminino	23	São Paulo	São Paulo	Sudeste	Ensino Superi	4	3	4	2	4	5 - Texto com muita coerência.	2	3	3	5 - Texto com muita coerência.
1252315829	12/11/2018	Masculino	43	Pirapora	Minas Gerai	Sudeste	Não Ensino Su	2	3	2	3	2	3	2	2	4	2
1253230369	09/11/2018	Feminino	30	São Paulo	São Paulo	Sudeste	Ensino Superi	5 - Muito simples.	2	5 - Muito simples.	3	5 - Muito simples.	5 - Texto com muita coerência.	3	5 - Texto com muita coerência.	1 - Texto sem coerência nenhuma	5 - Texto com muita coerência.
1254713915	12/11/2018	Feminino	46	Curitiba	Paraná	Sul	Ensino Superi	4	2	4	1- Muito complexo.	4	4	1 - Texto sem coerência nenhuma	5 - Texto com muita coerência.	1 - Texto sem coerência nenhuma	3
1256592490	12/11/2018	Masculino	36	Itambé	Bahia	Nordeste	Não Ensino Su	4	4	3	4	3	4	3	4	4	4
1256869206	09/11/2018	Masculino	32	São Paulo	São Paulo	Sudeste	Ensino Superi	4	2	4	1- Muito complexo.	4	3	2	5 - Texto com muita coerência.	1 - Texto sem coerência nenhuma	5 - Texto com muita coerência.
1259794752	12/11/2018	Feminino	36	Fortaleza	Ceará	Nordeste	Não Ensino Su	4	1- Muito complexo.	3	1- Muito complexo.	3	4	3	5 - Texto com muita coerência.	2	4
1259940770	12/11/2018	Masculino	17	Campinas	São Paulo	Sudeste	Não Ensino Su	1- Muito complexo.	1- Muito complexo.	4	1- Muito complexo.	1- Muito complexo.	1 - Texto sem coerência nenhuma	1 - Texto sem coerência nenhuma	3	1 - Texto sem coerência nenhuma	1 - Texto sem coerência nenhuma
1260065806	12/11/2018	Masculino	24	Santarém	Pará	Norte	Ensino Superi	3	2	5 - Muito simples.	1- Muito complexo.	3	5 - Texto com muita coerência.	5 - Texto com muita coerência.	5 - Texto com muita coerência.	1 - Texto sem coerência nenhuma	5 - Texto com muita coerência.
1261301725	11/11/2018	Feminino	25	Curitiba	Paraná	Sul	Ensino Superi	3	2	4	4	3	3	2	3	3	3
1262676841	10/11/2018	Feminino	46	Manaus	Amazonas	Norte	Ensino Superi	3	3	3	3	3	3	1 - Texto sem coerência nenhuma	3	2	3
1263913180	10/11/2018	Feminino	48	Pentecoste	Ceará	Nordeste	Ensino Superi	5 - Muito simples.	4	5 - Muito simples.	5 - Muito simples.	5 - Muito simples.	5 - Texto com muita coerência.	4	5 - Texto com muita coerência.	1 - Texto sem coerência nenhuma	5 - Texto com muita coerência.
1266395449	09/11/2018	Feminino	37	São Paulo	São Paulo	Sudeste	Ensino Superi	3	3	4	2	4	5 - Texto com muita coerência.	3	5 - Texto com muita coerência.	4	4
1267296220	12/11/2018	Masculino	37	Taubaté	São Paulo	Sudeste	Ensino Superi	3	1- Muito complexo.	3	1- Muito complexo.	3	3	3	3	3	3
1268394199	09/11/2018	Feminino	23	Lages	anta Catari	Sul	Ensino Superi	5 - Muito simples.	2	5 - Muito simples.	4	3	5 - Texto com muita coerência.	4	5 - Texto com muita coerência.	3	5 - Texto com muita coerência.
1268419117	09/11/2018	Feminino	49	Niterói	io de Janeir	Sudeste	Ensino Superi	1- Muito complexo.	1- Muito complexo.	1- Muito complexo.	1- Muito complexo.	1- Muito complexo.	5 - Texto com muita coerência.	3	3	3	3

Group B

User ID	Data	Gênero	Idade	Cidade	Estado	Região	Escolaridade	JUB- Em uma escala de um a cinco, onde 1 é muito complexo e 5 é muito simples, como você classificaria a complexidade do texto que acabou de ler?	RT1- Em uma escala de um a cinco, onde 1 é muito complexo e 5 é muito simples, como você classificaria a complexidade do texto que acabou de ler?	GAU- Em uma escala de um a cinco, onde 1 é muito complexo e 5 é muito simples, como você classificaria a complexidade do texto que acabou de ler?	MEL- Em uma escala de um a cinco, onde 1 é muito complexo e 5 é muito simples, como você classificaria a complexidade do texto que acabou de ler?	ST1- Em uma escala de um a cinco, onde 1 é muito complexo e 5 é muito simples, como você classificaria a complexidade do texto que acabou de ler?	JUB- Em uma escala de um a cinco, onde 1 é texto sem coerência nenhuma e 5 é texto com muita coerência, como você avalia a coerência do texto 1?	RT1- Em uma escala de um a cinco, onde 1 é texto sem coerência nenhuma e 5 é texto com muita coerência, como você avalia a coerência do texto 2?	GAU- Em uma escala de um a cinco, onde 1 é texto sem coerência nenhuma e 5 é texto com muita coerência, como você avalia a coerência do texto 3?	MEL- Em uma escala de um a cinco, onde 1 é texto sem coerência nenhuma e 5 é texto com muita coerência, como você avalia a coerência do texto 4?	ST1- Em uma escala de um a cinco, onde 1 é texto sem coerência nenhuma e 5 é texto com muita coerência, como você avalia a coerência do texto 6?
1001005843	12/11/201	Feminino	47	Brasília	Distrito Fe	Centro Oes	Ensino Superior	5 - Muito simples.	1- Muito complexo.	4	5 - Muito simples.	3	5 - Texto com muita coerência	1 - Texto sem coerência nenhuma	4	4	3
1001087037	09/11/201	Feminino	20	Curitiba	Paraná	Sul	Não Ensino Superior	3	2	3	3	2	4	3	5 - Texto com muita coerência	3	1 - Texto sem coerência nenhuma
1002286168	10/11/201	Feminino	43	Nilópolis	Rio de Jane	Sudeste	Ensino Superior	1- Muito complexo.	1- Muito complexo.	3	3	3	5 - Texto com muita coerência	2	1 - Texto sem coerência nenhuma	5 - Texto com muita coerência	4
1008770400	12/11/201	Feminino	65	Ribeirão Pi	São Paulo	Sudeste	Ensino Superior	5 - Muito simples.	1- Muito complexo.	3	5 - Muito simples.	5 - Muito simples.	5 - Texto com muita coerência	1 - Texto sem coerência nenhuma	2	5 - Texto com muita coerência	5 - Texto com muita coerência
1013560013	13/11/201	Feminino	37	Várzea Gra	Mato Gros	Centro Oes	Ensino Superior	4	1- Muito complexo.	1- Muito complexo.	2	3	4	1 - Texto sem coerência nenhuma	3	4	5 - Texto com muita coerência
1014047408	09/11/201	Masculino	43	Astorga	Paraná	Sul	Não Ensino Superior	4	1- Muito complexo.	4	1- Muito complexo.	5 - Muito simples.	5 - Texto com muita coerência	5 - Texto com muita coerência	5 - Texto com muita coerência	5 - Texto com muita coerência	5 - Texto com muita coerência
1014281607	09/11/201	Feminino	55	São Paulo	São Paulo	Sudeste	Ensino Superior	1- Muito complexo.	4	1- Muito complexo.	1- Muito complexo.	4	2	1 - Texto sem coerência nenhuma	5 - Texto com muita coerência	1 - Texto sem coerência nenhuma	5 - Texto com muita coerência
1017342333	09/11/201	Feminino	29	São Vicent	São Paulo	Sudeste	Ensino Superior	3	1- Muito complexo.	2	2	4	5 - Texto com muita coerência	1 - Texto sem coerência nenhuma	1 - Texto sem coerência nenhuma	2	5 - Texto com muita coerência
1018245950	12/11/201	Masculino	50	Nova Iguaç	Rio de Jane	Sudeste	Não Ensino Superior	3	1- Muito complexo.	1- Muito complexo.	5 - Muito simples.	1- Muito complexo.	1 - Texto sem coerência nenhuma	4	3	3	2
1018631962	09/11/201	Masculino	20	Caxias do S	Rio Grande	Sul	Ensino Superior	5 - Muito simples.	3	1- Muito complexo.	5 - Muito simples.	5 - Muito simples.	5 - Texto com muita coerência	2	1 - Texto sem coerência nenhuma	5 - Texto com muita coerência	5 - Texto com muita coerência
1025244089	09/11/201	Feminino	22	Curitiba	Paraná	Sul	Ensino Superior	5 - Muito simples.	1- Muito complexo.	3	4	5 - Muito simples.	5 - Texto com muita coerência	1 - Texto sem coerência nenhuma	3	5 - Texto com muita coerência	3
1027129351	11/11/201	Feminino	50	Pato Branc	Paraná	Sul	Ensino Superior	3	2	2	1- Muito complexo.	2	5 - Texto com muita coerência	1 - Texto sem coerência nenhuma	3	2	4
1028660245	09/11/201	Feminino	28	São José	Santa Cata	Sul	Não Ensino Superior	2	1- Muito complexo.	3	3	5 - Muito simples.	2	2	2	2	2
1029318810	10/11/201	Feminino	27	Salvador	Bahia	Nordeste	Ensino Superior	5 - Muito simples.	5 - Muito simples.	2	5 - Muito simples.	5 - Muito simples.	4	3	2	3	5 - Texto com muita coerência
1030598018	10/11/201	Masculino	26	Natal	Rio Grande	Nordeste	Ensino Superior	5 - Muito simples.	2	3	4	3	5 - Texto com muita coerência	1 - Texto sem coerência nenhuma	3	4	3
1031836843	12/11/201	Feminino	41	Crateús	Ceará	Nordeste	Não Ensino Superior	3	5 - Muito simples.	1- Muito complexo.	1- Muito complexo.	4	4	5 - Texto com muita coerência	4	5 - Texto com muita coerência	4
1032013220	11/11/201	Masculino	53	Bagé	Rio Grande	Sul	Ensino Superior	5 - Muito simples.	5 - Muito simples.	5 - Muito simples.	5 - Muito simples.	3	3	3	3	3	3
1034751007	09/11/201	Feminino	22	Bocaiúva	Minas Gera	Sudeste	Ensino Superior	3	3	1- Muito complexo.	2	2	5 - Texto com muita coerência	3	4	4	3
1035723095	10/11/201	Feminino	43	Castelo	Espírito Sa	Sudeste	Não Ensino Superior	1- Muito complexo.	1- Muito complexo.	1- Muito complexo.	1- Muito complexo.	5 - Muito simples.	2	2	2	3	5 - Texto com muita coerência
1036233129	11/11/201	Feminino	42	Jundiaí	São Paulo	Sudeste	Ensino Superior	3	1- Muito complexo.	3	2	4	4	3	5 - Texto com muita coerência	5 - Texto com muita coerência	4
1038757737	09/11/201	Masculino	39	São Gonçal	Rio Grande	Nordeste	Não Ensino Superior	1- Muito complexo.	1- Muito complexo.	1- Muito complexo.	2	5 - Muito simples.	5 - Texto com muita coerência	3	3	5 - Texto com muita coerência	2
1039074999	13/11/201	Feminino	44	Aracaju	Sergipe	Nordeste	Não Ensino Superior	5 - Muito simples.	1- Muito complexo.	1- Muito complexo.	3	5 - Muito simples.	5 - Texto com muita coerência	1 - Texto sem coerência nenhuma	3	3	5 - Texto com muita coerência
1039576514	09/11/201	Masculino	22	Aracaju	Sergipe	Nordeste	Ensino Superior	3	1- Muito complexo.	4	1- Muito complexo.	4	4	3	4	2	2
1040772238	09/11/201	Masculino	60	Belo Horizo	Minas Gera	Sudeste	Ensino Superior	2	1- Muito complexo.	1- Muito complexo.	1- Muito complexo.	2	5 - Texto com muita coerência	1 - Texto sem coerência nenhuma	3	3	3
1043673784	12/11/201	Feminino	38	Itaquaquec	São Paulo	Sudeste	Não Ensino Superior	1- Muito complexo.	2	2	3	3	3	4	5 - Texto com muita coerência	3	2
1044662774	09/11/201	Feminino	22	Salvador	Bahia	Nordeste	Ensino Superior	4	3	5 - Muito simples.	4	4	4	4	4	4	3
1049833171	09/11/201	Masculino	17	Monte Alto	São Paulo	Sudeste	Não Ensino Superior	4	1- Muito complexo.	1- Muito complexo.	1- Muito complexo.	3	5 - Texto com muita coerência	3	4	3	5 - Texto com muita coerência
1051144239	10/11/201	Feminino	37	Vitória da (	Bahia	Nordeste	Não Ensino Superior	3	2	1- Muito complexo.	3	4	3	1 - Texto sem coerência nenhuma	2	2	5 - Texto com muita coerência
1053254874	12/11/201	Feminino	38	Olinda	Pernambu	Nordeste	Não Ensino Superior	4	3	1- Muito complexo.	2	5 - Muito simples.	3	3	3	3	5 - Texto com muita coerência
1053617742	10/11/201	Feminino	27	Itapetining	São Paulo	Sudeste	Não Ensino Superior	2	1- Muito complexo.	2	1- Muito complexo.	2	5 - Texto com muita coerência	3	5 - Texto com muita coerência	3	5 - Texto com muita coerência
1054479165	12/11/201	Masculino	38	Poá	São Paulo	Sudeste	Não Ensino Superior	3	2	2	3	3	3	3	3	3	3
1055462804	09/11/201	Masculino	38	Petrópolis	Rio de Jane	Sudeste	Ensino Superior	3	3	3	3	5 - Muito simples.	3	4	3	4	4
1056391445	09/11/201	Feminino	21	Perdigão	Minas Gera	Sudeste	Ensino Superior	5 - Muito simples.	4	2	4	5 - Muito simples.	5 - Texto com muita coerência	3	3	2	4
1056956313	09/11/201	Feminino	21	Porto Velh	Rondônia	Norte	Ensino Superior	3	1- Muito complexo.	1- Muito complexo.	2	5 - Muito simples.	5 - Texto com muita coerência	1 - Texto sem coerência nenhuma	2	5 - Texto com muita coerência	3
1057808772	11/11/201	Feminino	41	Salvador	Bahia	Nordeste	Ensino Superior	4	1- Muito complexo.	2	3	4	3	1 - Texto sem coerência nenhuma	2	3	4
1059143124	09/11/201	Feminino	38	Cruzeiro	São Paulo	Sudeste	Ensino Superior	3	1- Muito complexo.	2	4	4	4	2	2	4	3
1060106951	09/11/201	Feminino	33	Salvador	Bahia	Nordeste	Não Ensino Superior	3	3	3	3	3	3	3	3	3	4
1061132886	09/11/201	Masculino	33	João Pesso	Paraíba	Nordeste	Ensino Superior	4	5 - Muito simples.	2	3	5 - Muito simples.	5 - Texto com muita coerência	5 - Texto com muita coerência	5 - Texto com muita coerência	5 - Texto com muita coerência	4
1061994965	12/11/201	Feminino	38	Mauá	São Paulo	Sudeste	Não Ensino Superior	1- Muito complexo.	1- Muito complexo.	2	3	1- Muito complexo.	4	3	3	2	3
1065324348	10/11/201	Feminino	43	Fortaleza	Ceará	Nordeste	Não Ensino Superior	2	3	3	4	3	4	4	4	4	4
1066920957	09/11/201	Masculino	66	Rio de Jane	Rio de Jane	Sudeste	Ensino Superior	5 - Muito simples.	1- Muito complexo.	2	4	3	5 - Texto com muita coerência	1 - Texto sem coerência nenhuma	2	4	5 - Texto com muita coerência
1069067849	09/11/201	Masculino	38	Salvador	Bahia	Nordeste	Ensino Superior	4	4	3	3	3	4	3	4	3	3
1069780648	09/11/201	Feminino	32	Ananindeu	Pará	Norte	Não Ensino Superior	2	1- Muito complexo.	1- Muito complexo.	3	2	4	2	2	1 - Texto sem coerência nenhuma	5 - Texto com muita coerência
1070815166	09/11/201	Masculino	41	São José do	Paraná	Sul	Não Ensino Superior	2	2	3	3	3	4	2	3	3	4
1074711165	12/11/201	Masculino	25	Montes Cla	Minas Gera	Sudeste	Ensino Superior	1- Muito complexo.	1- Muito complexo.	1- Muito complexo.	2	2	3	5 - Texto com muita coerência	3	4	1 - Texto sem coerência nenhuma
1074901282	09/11/201	Masculino	18	Brasília	Distrito Fe	Centro Oes	Não Ensino Superior	2	3	2	5 - Muito simples.	2	3	3	3	3	3
1076086042	09/11/201	Masculino	27	São João d	Goiás	Centro Oes	Ensino Superior	4	1- Muito complexo.	1- Muito complexo.	4	5 - Muito simples.	4	1 - Texto sem coerência nenhuma	2	4	5 - Texto com muita coerência
1076660998	10/11/201	Feminino	20	Entre Rios	Bahia	Nordeste	Não Ensino Superior	3	1- Muito complexo.	3	5 - Muito simples.	4	2	1 - Texto sem coerência nenhuma	3	4	3
1079061122	12/11/201	Masculino	38	Salvador	Bahia	Nordeste	Ensino Superior	4	2	2	4	5 - Muito simples.	5 - Texto com muita coerência	1 - Texto sem coerência nenhuma	4	5 - Texto com muita coerência	5 - Texto com muita coerência
1079129759	12/11/201	Masculino	39	Limeira	São Paulo	Sudeste	Não Ensino Superior	4	1- Muito complexo.	2	3	4	3	3	2	1 - Texto sem coerência nenhuma	4
1080794053	09/11/201	Masculino	61	Itapevi	São Paulo	Sudeste	Ensino Superior	5 - Muito simples.	1- Muito complexo.	3	4	5 - Muito simples.	5 - Texto com muita coerência	2	5 - Texto com muita coerência	4	5 - Texto com muita coerência
1081774756	12/11/201	Feminino	49	Cariacica	Espírito Sa	Sudeste	Não Ensino Superior	4	3	1- Muito complexo.	4	5 - Muito simples.	1 - Texto sem coerência nenhuma	2	4	4	1 - Texto sem coerência nenhuma
1084697328	09/11/201	Masculino	22	Jaboatão d	Pernambu	Nordeste	Ensino Superior	5 - Muito simples.	3	2	5 - Muito simples.	5 - Muito simples.	5 - Texto com muita coerência	1 - Texto sem coerência nenhuma	3	2	5 - Texto com muita coerência
1085198917	09/11/201	Feminino	39	São José do	São Paulo	Sudeste	Não Ensino Superior	1- Muito complexo.	2	4	1- Muito complexo.	3	3	4	2	3	4
1085422326	09/11/201	Masculino	42	Fortaleza	Ceará	Nordeste	Ensino Superior	3	1- Muito complexo.	2	5 - Muito simples.	3	4	3	2	3	5 - Texto com muita coerência
1086705794	12/11/201	Feminino	37	Várzea Gra	Mato Gros	Centro Oes	Não Ensino Superior	1- Muito complexo.	5 - Muito simples.	1- Muito complexo.	1- Muito complexo.	1- Muito complexo.	5 - Texto com muita coerência	5 - Texto com muita coerência	5 - Texto com muita coerência	5 - Texto com muita coerência	5 - Texto com muita coerência
1087024848	10/11/201	Masculino	20	Santos	São Paulo	Sudeste	Não Ensino Superior	2	5 - Muito simples.	5 - Muito simples.	1- Muito complexo.	5 - Muito simples.	5 - Texto com muita coerência	3	1 - Texto sem coerência nenhuma	5 - Texto com muita coerência	1 - Texto sem coerência nenhuma
1087776627	09/11/201	Masculino	41	Curitiba	Paraná	Sul	Ensino Superior	3	2	2	3	5 - Muito simples.	3	4	2	4	5 - Texto com muita coerência
1088231385	09/11/201	Masculino	18	Diamantina	Minas Gera	Sudeste	Ensino Superior	4	3	2	2	5 - Muito simples.	4	2	3	4	5 - Texto com muita coerência
1088956474	09/11/201	Feminino	33	Araçatuba	São Paulo	Sudeste	Ensino Superior	5 - Muito simples.	1- Muito complexo.	4	4	5 - Muito simples.	5 - Texto com muita coerência	1 - Texto sem coerência nenhuma	5 - Texto com muita coerência	4	4
1089471668	09/11/201	Feminino	21	Várzea Gra	Mato Gros	Centro Oes	Ensino Superior	4	1- Muito complexo.	2	4	5 - Muito simples.	5 - Texto com muita coerência	1 - Texto sem coerência nenhuma	3	5 - Texto com muita coerência	4
1090373408	09/11/201	Feminino	20	Santo Andr	São Paulo	Sudeste	Não Ensino Superior	4	1- Muito complexo.	1- Muito complexo.	3	3	4	2	3	4	3
1091028183	12/11/201	Masculino	51	Santo Andr	São Paulo	Sudeste	Não Ensino Superior	3	2	3	3	3	3	5 - Texto com muita coerência	2	4	3
1091093106	12/11/201	Feminino	36	Palmeira d	Rio Grande	Sul	Não Ensino Superior	2	1- Muito complexo.	1- Muito complexo.	2	2	4	4	4	4	4
1094143256	09/11/201	Masculino	37	São Paulo	São Paulo	Sudeste	Ensino Superior	3	2	2	3	3	3	2	3	3	4
1094515475	09/11/201	Feminino	27	Poá	São Paulo	Sudeste	Ensino Superior	4	2	1- Muito complexo.	3	3	3	3	3	4	3
1097809263	13/11/201	Feminino	40	Sorocaba	São Paulo	Sudeste	Não Ensino Superior	3	1- Muito complexo.	1- Muito complexo.	2	3	4	1 - Texto sem coerência nenhuma	3	2	4
1098385572	09/11/201	Feminino	32	Brasília	Distrito Fe	Centro Oes	Ensino Superior	4	1- Muito complexo.	1- Muito complexo.	3	3	5 - Texto com muita coerência	1 - Texto sem coerência nenhuma	3	4	5 - Texto com muita coerência
1100976737	09/11/201	Masculino	24	Campo Gra	Mato Gros	Centro Oes	Ensino Superior	3	2	1- Muito complexo.	4	4	5 - Texto com muita coerência	2	4	5 - Texto com muita coerência	5 - Texto com muita coerência
1103612146	10/11/201	Feminino	24	São Paulo	São Paulo	Sudeste	Ensino Superior	3	3	3	5 - Muito simples.	5 - Muito simples.	5 - Texto com muita coerência	4	3	5 - Texto com muita coerência	5 - Texto com muita coerência
1104233743	09/11/201	Masculino	19	Pindamonh	São Paulo	Sudeste	Ensino Superior	3	2	1- Muito complexo.	4	4	4	3	5 - Texto com muita coerência	5 - Texto com muita coerência	5 - Texto com muita coerência
1104537538	09/11/201	Masculino	50	Porto Aleg	Rio Grande	Sul	Não Ensino Superior	5 - Muito simples.	3	4	3	5 - Muito simples.	2	1 - Texto sem coerência nenhuma	1 - Texto sem coerência nenhuma	1 - Texto sem coerência nenhuma	3
1105326195	12/11/201	Feminino	47	Guareí	São Paulo	Sudeste	Não Ensino Superior	3	3	3	3	3	2	3	3	3	2
1106331101	09/11/201	Feminino	46	Registro	São Paulo	Sudeste	Ensino Superior	5 - Muito simples.	1- Muito complexo.	1- Muito complexo.	1- Muito complexo.	4	5 - Texto com muita coerência	1 - Texto sem coerência nenhuma	2	3	5 - Texto com muita coerência
1110067020	10/11/201	Feminino	44	Salvador	Bahia	Nordeste	Ensino Superior	4	3	3	4	3	4	3	3	3	4
1110714599	10/11/201	Masculino	22	Altinho	Pernambu	Nordeste	Ensino Superior	4	2	1- Muito complexo.	3	4	5 - Texto com muita coerência	2	2	3	5 - Texto com muita coerência
1110800461	09/11/201	Feminino	26	Araçoiaba (	São Paulo	Sudeste	Ensino Superior	5 - Muito simples.	1- Muito complexo.	3	5 - Muito simples.	2	3	3	3	3	5 - Texto com muita coerência
1113622172	09/11/201	Masculino	43	Tamandaré	Pernambu	Nordeste	Ensino Superior	2	1- Muito complexo.	3	4	5 - Muito simples.	1 - Texto sem coerência nenhuma	1 - Texto sem coerência nenhuma	4	1 - Texto sem coerência nenhuma	1 - Texto sem coerência nenhuma
1113715836	09/11/201	Masculino	28	São Mateu	Maranhão	Nordeste	Não Ensino Superior	4	3	4	4	3	4	3	4	3	4
1113922514	09/11/201	Feminino	21	Caucaia	Ceará	Nordeste	Não Ensino Superior	4	1- Muito complexo.	3	2	5 - Muito simples.	5 - Texto com muita coerência	1 - Texto sem coerência nenhuma	3	4	2
1119910398	12/11/201	Feminino	22	Fortaleza	Ceará	Nordeste	Ensino Superior	2	1- Muito complexo.	1- Muito complexo.	1- Muito complexo.	4	2	1 - Texto sem coerência nenhuma	3	2	4

1120126018	12/11/201	Feminino	30	Fortaleza	Ceará	Nordeste	Não Ensino Superior	2	3	2	3	4	5 - Texto com muita coerência	3	2	4	4
1121430159	12/11/201	Masculino	19	Juazeiro	Bahia	Nordeste	Ensino Superior	3	2	3	3	2	2	5 - Texto com muita coerência	2	3	2
1122122256	09/11/201	Masculino	26	Salgueiro	Pernambu	Nordeste	Não Ensino Superior	1- Muito complexo.	3	1- Muito complexo.	1- Muito complexo.	5 - Muito simples.	1 - Texto sem coerência nenhuma	1 - Texto sem coerência nenhuma	1 - Texto sem coerência nenhuma	5 - Texto com muita coerência	5 - Texto com muita coerência
1126635704	10/11/201	Feminino	30	Suzano	São Paulo	Sudeste	Não Ensino Superior	5 - Muito simples.	5 - Muito simples.	1- Muito complexo.	1- Muito complexo.	5 - Muito simples.	5 - Texto com muita coerência	1 - Texto sem coerência nenhuma	5 - Texto com muita coerência	5 - Texto com muita coerência	4
1126767617	09/11/201	Feminino	39	Curitiba	Paraná	Sul	Não Ensino Superior	4	1- Muito complexo.	3	3	5 - Muito simples.	4	1 - Texto sem coerência nenhuma	3	3	4
1127840384	11/11/201	Feminino	31	Curitiba	Paraná	Sul	Ensino Superior	5 - Muito simples.	1- Muito complexo.	3	3	4	5 - Texto com muita coerência	1 - Texto sem coerência nenhuma	3	3	2
1128219951	10/11/201	Feminino	38	Eusébio	Ceará	Nordeste	Ensino Superior	5 - Muito simples.	4	3	4	5 - Muito simples.	4	3	3	3	4
1128710684	12/11/201	Feminino	37	São Paulo	São Paulo	Sudeste	Ensino Superior	3	1- Muito complexo.	1- Muito complexo.	5 - Muito simples.	5 - Muito simples.	5 - Texto com muita coerência	1 - Texto sem coerência nenhuma	2	4	5 - Texto com muita coerência
1129340531	11/11/201	Masculino	22	Dourados	Mato Gros	Centro Oes	Ensino Superior	4	2	2	2	3	3	3	3	3	4
1131439814	11/11/201	Feminino	29	São José d	Paraná	Sul	Não Ensino Superior	5 - Muito simples.	3	2	5 - Muito simples.	5 - Muito simples.	3	1 - Texto sem coerência nenhuma	2	3	4
1132128428	10/11/201	Feminino	30	Indaiatuba	São Paulo	Sudeste	Ensino Superior	3	3	3	3	3	3	3	3	3	3
1132333374	10/11/201	Feminino	28	Rio de Jane	Rio de Jane	Sudeste	Ensino Superior	4	1- Muito complexo.	1- Muito complexo.	4	5 - Muito simples.	4	2	2	3	4
1132342880	11/11/201	Feminino	28	Nova Vené	Espírito Sa	Sudeste	Ensino Superior	5 - Muito simples.	2	2	5 - Muito simples.	5 - Muito simples.	5 - Texto com muita coerência	4	1 - Texto sem coerência nenhuma	5 - Texto com muita coerência	5 - Texto com muita coerência
1135668838	09/11/201	Masculino	36	São José d	Paraná	Sul	Não Ensino Superior	1- Muito complexo.	3	3	2	2	2	1 - Texto sem coerência nenhuma	1 - Texto sem coerência nenhuma	3	5 - Texto com muita coerência
1137232625	09/11/201	Masculino	41	Belo Horiz	Minas Gera	Sudeste	Ensino Superior	3	3	3	3	3	3	3	1 - Texto sem coerência nenhuma	4	3
1138158413	09/11/201	Masculino	36	Sorriso	Mato Gros	Centro Oes	Não Ensino Superior	5 - Muito simples.	4	5 - Muito simples.	4	5 - Muito simples.	3	1 - Texto sem coerência nenhuma	1 - Texto sem coerência nenhuma	5 - Texto com muita coerência	1 - Texto sem coerência nenhuma
1140900564	11/11/201	Masculino	19	Sapucaia d	Rio Grande	Sul	Ensino Superior	5 - Muito simples.	1- Muito complexo.	3	5 - Muito simples.	5 - Muito simples.	5 - Texto com muita coerência	1 - Texto sem coerência nenhuma	3	5 - Texto com muita coerência	5 - Texto com muita coerência
1143785437	09/11/201	Feminino	25	Belo Horiz	Minas Gera	Sudeste	Ensino Superior	5 - Muito simples.	1- Muito complexo.	1- Muito complexo.	3	5 - Muito simples.	1 - Texto sem coerência nenhuma	1 - Texto sem coerência nenhuma	5 - Texto com muita coerência	3	5 - Texto com muita coerência
1144914997	09/11/201	Masculino	45	Santa Bárb	Minas Gera	Sudeste	Não Ensino Superior	3	3	3	3	3	3	4	3	4	4
1145204176	13/11/201	Masculino	40	Porto Aleg	Rio Grande	Sul	Ensino Superior	3	3	3	3	4	4	3	3	4	3
1145822822	09/11/201	Masculino	36	Ilhota	Santa Cata	Sul	Não Ensino Superior	4	1- Muito complexo.	1- Muito complexo.	3	5 - Muito simples.	5 - Texto com muita coerência	1 - Texto sem coerência nenhuma	3	4	5 - Texto com muita coerência
1146237393	09/11/201	Feminino	23	Rio de Jane	Rio de Jane	Sudeste	Não Ensino Superior	5 - Muito simples.	3	2	1- Muito complexo.	5 - Muito simples.	1 - Texto sem coerência nenhuma	5 - Texto com muita coerência	1 - Texto sem coerência nenhuma	5 - Texto com muita coerência	1 - Texto sem coerência nenhuma
1146800958	10/11/201	Feminino	60	Santa Isabe	São Paulo	Sudeste	Ensino Superior	4	2	4	3	5 - Muito simples.	3	4	3	4	3
1147637764	10/11/201	Masculino	28	Natal	Rio Grande	Nordeste	Ensino Superior	5 - Muito simples.	1- Muito complexo.	1- Muito complexo.	5 - Muito simples.	5 - Muito simples.	5 - Texto com muita coerência	2	3	4	5 - Texto com muita coerência
1147778845	12/11/201	Masculino	42	São Paulo	São Paulo	Sudeste	Ensino Superior	4	2	3	3	3	4	2	2	5 - Texto com muita coerência	5 - Texto com muita coerência
1148348587	09/11/201	Feminino	46	Terra Boa	Paraná	Sul	Não Ensino Superior	1- Muito complexo.	1- Muito complexo.	1- Muito complexo.	1- Muito complexo.	1- Muito complexo.	4	3	4	4	3
1150635500	10/11/201	Feminino	20	São José d	São Paulo	Sudeste	Ensino Superior	3	1- Muito complexo.	3	4	5 - Muito simples.	5 - Texto com muita coerência	1 - Texto sem coerência nenhuma	3	4	5 - Texto com muita coerência
1150733286	13/11/201	Feminino	54	Juiz de For	Minas Gera	Sudeste	Não Ensino Superior	3	4	3	3	4	4	4	2	3	3
1151047929	11/11/201	Feminino	26	Palmas	Tocantins	Norte	Ensino Superior	4	3	3	4	5 - Muito simples.	5 - Texto com muita coerência	1 - Texto sem coerência nenhuma	4	5 - Texto com muita coerência	4
1152035882	10/11/201	Masculino	27	Pombal	Paraíba	Nordeste	Não Ensino Superior	3	2	2	4	3	4	2	2	3	4
1152478548	12/11/201	Masculino	57	Piracicaba	São Paulo	Sudeste	Ensino Superior	4	4	4	4	4	4	4	4	4	3
1153347613	09/11/201	Masculino	19	Belém	Pará	Norte	Não Ensino Superior	3	1- Muito complexo.	2	3	4	4	3	3	3	2
1156507860	09/11/201	Masculino	18	Castanhal	Pará	Norte	Ensino Superior	3	1- Muito complexo.	3	2	5 - Muito simples.	4	1 - Texto sem coerência nenhuma	3	4	5 - Texto com muita coerência
1158005468	09/11/201	Masculino	31	Brasília	Distrito Fe	Centro Oes	Ensino Superior	3	3	3	4	4	3	3	3	4	3
1158063216	09/11/201	Feminino	42	Santo Andr	São Paulo	Sudeste	Não Ensino Superior	3	4	4	3	3	3	4	3	3	3
1159349613	13/11/201	Feminino	18	Guarulhos	São Paulo	Sudeste	Não Ensino Superior	2	1- Muito complexo.	2	3	3	3	3	4	3	4
1160255894	09/11/201	Feminino	40	Itaquaque	São Paulo	Sudeste	Ensino Superior	3	1- Muito complexo.	2	2	4	4	3	3	4	4
1164922553	09/11/201	Masculino	23	Demerval L	Piauí	Nordeste	Ensino Superior	3	2	3	5 - Muito simples.	3	3	3	3	3	3
1167477214	11/11/201	Feminino	52	Belo Horiz	Minas Gera	Sudeste	Ensino Superior	2	1- Muito complexo.	1- Muito complexo.	3	4	4	4	3	4	4
1168093386	09/11/201	Masculino	36	Belém	Pará	Norte	Não Ensino Superior	3	4	4	3	4	3	4	4	3	2
1172605105	09/11/201	Masculino	21	Apodi	Rio Grande	Nordeste	Ensino Superior	3	2	1- Muito complexo.	2	3	4	3	3	3	2
1173699986	12/11/201	Feminino	23	Lavras	Minas Gera	Sudeste	Ensino Superior	5 - Muito simples.	1- Muito complexo.	1- Muito complexo.	3	5 - Muito simples.	5 - Texto com muita coerência	2	4	4	3
1177621573	09/11/201	Feminino	34	Aracaju	Sergipe	Nordeste	Ensino Superior	5 - Muito simples.	1- Muito complexo.	2	2	5 - Muito simples.	5 - Texto com muita coerência	1 - Texto sem coerência nenhuma	5 - Texto com muita coerência	5 - Texto com muita coerência	5 - Texto com muita coerência
1177835888	12/11/201	Feminino	39	Rio de Jane	Rio de Jane	Sudeste	Não Ensino Superior	3	2	4	5 - Muito simples.	2	2	3	2	3	3
1178740243	09/11/201	Masculino	41	Fazenda Ri	Paraná	Sul	Não Ensino Superior	3	4	2	3	5 - Muito simples.	2	4	4	3	5 - Texto com muita coerência
1179117685	09/11/201	Masculino	33	São Paulo	São Paulo	Sudeste	Ensino Superior	3	3	3	3	5 - Muito simples.	3	3	3	3	3
1179329836	09/11/201	Feminino	27	São Paulo	São Paulo	Sudeste	Ensino Superior	4	1- Muito complexo.	2	5 - Muito simples.	5 - Muito simples.	5 - Texto com muita coerência	4	5 - Texto com muita coerência	5 - Texto com muita coerência	5 - Texto com muita coerência
1179435592	09/11/201	Masculino	27	Cuité	Paraíba	Nordeste	Não Ensino Superior	5 - Muito simples.	3	3	3	3	3	3	3	3	3
1185015256	09/11/201	Feminino	36	Planaltina	Goiás	Centro Oes	Não Ensino Superior	1- Muito complexo.	1- Muito complexo.	1- Muito complexo.	1- Muito complexo.	1- Muito complexo.	2	2	2	2	2
1185246979	10/11/201	Masculino	42	Mauá	São Paulo	Sudeste	Ensino Superior	3	1- Muito complexo.	2	2	3	3	2	2	3	4
1185485994	09/11/201	Feminino	36	São Paulo	São Paulo	Sudeste	Ensino Superior	3	1- Muito complexo.	1- Muito complexo.	2	4	4	1 - Texto sem coerência nenhuma	2	3	5 - Texto com muita coerência
1185785481	09/11/201	Feminino	58	Curitiba	Paraná	Sul	Ensino Superior	4	1- Muito complexo.	1- Muito complexo.	1- Muito complexo.	4	5 - Texto com muita coerência	1 - Texto sem coerência nenhuma	3	4	5 - Texto com muita coerência
1185858702	09/11/201	Feminino	17	Brasília	Distrito Fe	Centro Oes	Não Ensino Superior	3	3	3	3	3	3	3	3	3	3
1189660041	10/11/201	Feminino	26	Maracanaú	Ceará	Nordeste	Ensino Superior	4	1- Muito complexo.	2	5 - Muito simples.	5 - Muito simples.	3	1 - Texto sem coerência nenhuma	3	5 - Texto com muita coerência	5 - Texto com muita coerência
1189816474	10/11/201	Feminino	31	Cabo de Sa	Pernambu	Nordeste	Não Ensino Superior	4	1- Muito complexo.	1- Muito complexo.	4	3	4	2	5 - Texto com muita coerência	3	5 - Texto com muita coerência
1190809687	10/11/201	Masculino	18	Porto da F	Sergipe	Nordeste	Não Ensino Superior	5 - Muito simples.	1- Muito complexo.	3	4	5 - Muito simples.	5 - Texto com muita coerência	2	4	5 - Texto com muita coerência	5 - Texto com muita coerência
1191914546	12/11/201	Masculino	42	Itapevi	São Paulo	Sudeste	Ensino Superior	3	1- Muito complexo.	1- Muito complexo.	1- Muito complexo.	4	5 - Texto com muita coerência	2	3	3	5 - Texto com muita coerência
1192816678	12/11/201	Masculino	21	São Gonçal	Rio de Jane	Sudeste	Ensino Superior	3	3	3	3	3	3	3	3	3	3
1193182933	12/11/201	Feminino	20	Brasília	Distrito Fe	Centro Oes	Ensino Superior	4	3	3	4	5 - Muito simples.	5 - Texto com muita coerência	4	3	5 - Texto com muita coerência	3
1194935925	10/11/201	Masculino	38	Encruzilha	Bahia	Nordeste	Ensino Superior	5 - Muito simples.	1- Muito complexo.	1- Muito complexo.	2	4	3	1 - Texto sem coerência nenhuma	4	5 - Texto com muita coerência	5 - Texto com muita coerência
1194959235	12/11/201	Masculino	36	Gameleiras	Minas Gera	Sudeste	Não Ensino Superior	2	1- Muito complexo.	2	3	2	4	4	4	4	4
1195072353	09/11/201	Masculino	39	Mauá	São Paulo	Sudeste	Ensino Superior	3	2	2	2	2	2	3	2	2	2
1195223336	09/11/201	Feminino	36	Balneário (	Santa Cata	Sul	Não Ensino Superior	3	1- Muito complexo.	1- Muito complexo.	5 - Muito simples.	5 - Muito simples.	4	1 - Texto sem coerência nenhuma	1 - Texto sem coerência nenhuma	1 - Texto sem coerência nenhuma	2
1195253077	12/11/201	Masculino	47	Piracaia	São Paulo	Sudeste	Ensino Superior	5 - Muito simples.	1- Muito complexo.	2	4	4	5 - Texto com muita coerência	1 - Texto sem coerência nenhuma	5 - Texto com muita coerência	5 - Texto com muita coerência	5 - Texto com muita coerência
1197530692	09/11/201	Masculino	42	Salvador	Bahia	Nordeste	Ensino Superior	3	1- Muito complexo.	2	2	3	3	3	4	3	4
1200706645	11/11/201	Feminino	37	Rio de Jane	Rio de Jane	Sudeste	Não Ensino Superior	5 - Muito simples.	3	5 - Muito simples.	3	2	5 - Texto com muita coerência	2	4	3	3
1201677853	12/11/201	Feminino	39	Rio de Jane	Rio de Jane	Sudeste	Não Ensino Superior	1- Muito complexo.	3	3	3	3	2	2	3	3	3
1201800447	09/11/201	Feminino	27	Rio de Jane	Rio de Jane	Sudeste	Ensino Superior	5 - Muito simples.	1- Muito complexo.	3	4	5 - Muito simples.	5 - Texto com muita coerência	1 - Texto sem coerência nenhuma	3	5 - Texto com muita coerência	5 - Texto com muita coerência
1204250480	10/11/201	Feminino	26	Santarém	Pará	Norte	Ensino Superior	5 - Muito simples.	3	3	3	5 - Muito simples.	5 - Texto com muita coerência	1 - Texto sem coerência nenhuma	5 - Texto com muita coerência	5 - Texto com muita coerência	5 - Texto com muita coerência
1204694449	09/11/201	Feminino	46	Brasília	Distrito Fe	Centro Oes	Ensino Superior	2	4	3	5 - Muito simples.	3	3	2	3	3	4
1204900621	09/11/201	Masculino	31	Várzea da I	Minas Gera	Sudeste	Não Ensino Superior	3	1- Muito complexo.	1- Muito complexo.	3	2	2	2	5 - Texto com muita coerência	5 - Texto com muita coerência	3
1205048806	10/11/201	Feminino	26	São Paulo	São Paulo	Sudeste	Ensino Superior	3	1- Muito complexo.	4	3	5 - Muito simples.	4	1 - Texto sem coerência nenhuma	4	4	5 - Texto com muita coerência
1207176648	10/11/201	Feminino	18	Campos dc	Rio de Jane	Sudeste	Não Ensino Superior	3	1- Muito complexo.	3	3	5 - Muito simples.	4	1 - Texto sem coerência nenhuma	2	3	3
1208088161	09/11/201	Feminino	50	Pariquera-	São Paulo	Sudeste	Não Ensino Superior	3	1- Muito complexo.	3	2	5 - Muito simples.	3	3	2	3	4
1212366100	12/11/201	Feminino	32	São Paulo	São Paulo	Sudeste	Ensino Superior	4	1- Muito complexo.	2	3	5 - Muito simples.	5 - Texto com muita coerência	1 - Texto sem coerência nenhuma	4	4	5 - Texto com muita coerência
1212384690	09/11/201	Masculino	28	Imbé	Rio Grande	Sul	Não Ensino Superior	3	1- Muito complexo.	2	2	1- Muito complexo.	4	2	3	3	5 - Texto com muita coerência
1215579308	09/11/201	Feminino	37	Rio de Jane	Rio de Jane	Sudeste	Ensino Superior	2	1- Muito complexo.	1- Muito complexo.	3	3	5 - Texto com muita coerência	3	3	5 - Texto com muita coerência	5 - Texto com muita coerência
1216798271	09/11/201	Masculino	19	Oiapoque	Amapá	Norte	Ensino Superior	2	3	4	3	5 - Muito simples.	3	2	3	3	2
1217491199	10/11/201	Masculino	38	Goiânia	Goiás	Centro Oes	Ensino Superior	5 - Muito simples.	1- Muito complexo.	1- Muito complexo.	3	2	3	1 - Texto sem coerência nenhuma	3	3	3
1217803089	12/11/201	Masculino	52	Cariacica	Espírito Sa	Sudeste	Não Ensino Superior	3	1- Muito complexo.	5 - Muito simples.	1- Muito complexo.	5 - Muito simples.	1 - Texto sem coerência nenhuma	1 - Texto sem coerência nenhuma	5 - Texto com muita coerência	5 - Texto com muita coerência	3
1218179088	09/11/201	Feminino	19	Belo Horiz	Minas Gera	Sudeste	Ensino Superior	4	1- Muito complexo.	1- Muito complexo.	2	4	5 - Texto com muita coerência	3	3	5 - Texto com muita coerência	5 - Texto com muita coerência
1219574875	09/11/201	Feminino	53	Mogi das C	São Paulo	Sudeste	Não Ensino Superior	4	4	4	4	4	3	3	4	4	3
1220350630	12/11/201	Feminino	49	Duque de (	Rio de Jane	Sudeste	Não Ensino Superior	3	1- Muito complexo.	1- Muito complexo.	2	4	5 - Texto com muita coerência	1 - Texto sem coerência nenhuma	1 - Texto sem coerência nenhuma	4	5 - Texto com muita coerência
1221834603	09/11/201	Masculino	27	Osasco	São Paulo	Sudeste	Ensino Superior	4	3	4	4	5 - Muito simples.	4	4	3	4	3
1223945380	09/11/201	Feminino	25	São Paulo	São Paulo	Sudeste	Ensino Superior	5 - Muito simples.	1- Muito complexo.	3	4	5 - Muito simples.	5 - Texto com muita coerência	1 - Texto sem coerência nenhuma	4	5 - Texto com muita coerência	5 - Texto com muita coerência
1224248718	09/11/201	Feminino	33	Ceilândia	Distrito Fe	Centro Oes	Não Ensino Superior	4	1- Muito complexo.	2	3	5 - Muito simples.	4	1 - Texto sem coerência nenhuma	2	5 - Texto com muita coerência	5 - Texto com muita coerência
1224653773	12/11/201	Feminino	36	Arraial do (	Rio de Jane	Sudeste	Ensino Superior	5 - Muito simples.	5 - Muito simples.	5 - Muito simples.	5 - Muito simples.	5 - Muito simples.	5 - Texto com muita coerência	5 - Texto com muita coerência	5 - Texto com muita coerência	5 - Texto com muita coerência	5 - Texto com muita coerência
1225996144	10/11/201	Masculino	41	Santa Luzia	Paraíba	Nordeste	Ensino Superior	2	2	1- Muito complexo.	3	2	3	4	2	3	2
1227903743	10/11/201	Feminino	23	Catalão	Goiás	Centro Oes	Ensino Superior	5 - Muito simples.	1- Muito complexo.	1- Muito complexo.	3	2	3	2	3	3	3
1229393796	09/11/201	Masculino	17	Catunda	Ceará	Nordeste	Não Ensino Superior	2	1- Muito complexo.	1- Muito complexo.	3	5 - Muito simples.	5 - Texto com muita coerência	3	3	5 - Texto com muita coerência	5 - Texto com muita coerência
1231665948	12/11/201	Feminino	38	Guarulhos	São Paulo	Sudeste	Não Ensino Superior	3	3	3	3	2	3	2	3	3	3
1232209668	12/11/201	Masculino	36	Rio de Jane	Rio de Jane	Sudeste	Não Ensino Superior	2	1- Muito complexo.	2	1- Muito complexo.	2	5 - Texto com muita coerência	3	2	5 - Texto com muita coerência	5 - Texto com muita coerência
1234137577	10/11/201	Feminino	36	Sinop	Mato Gros	Centro Oes	Ensino Superior	4	1- Muito complexo.	2	4	4	5 - Texto com muita coerência	2	4	5 - Texto com muita coerência	5 - Texto com muita coerência

1234235886	09/11/201	Feminino	28	Marabá	Pará	Norte	Ensino Superior	2	2	2	3	4	5 - Texto com muita coerência	4	3	2	4
1237266112	09/11/201	Feminino	47	Fortaleza	Ceará	Nordeste	Não Ensino Superior	5 - Muito simples.	4	4	3	5 - Muito simples.	3	4	5 - Texto com muita coerência	3	4
1239199246	10/11/201	Masculino	37	Duque de (	Rio de Jane	Sudeste	Não Ensino Superior	5 - Muito simples.	5 - Muito simples.	1- Muito complexo.	1- Muito complexo.	1- Muito complexo.	1 - Texto sem coerência nenhuma	5 - Texto com muita coerência	3	4	5 - Texto com muita coerência
1239713003	09/11/201	Masculino	33	Itapevi	São Paulo	Sudeste	Ensino Superior	5 - Muito simples.	2	3	3	5 - Muito simples.	5 - Texto com muita coerência	3	5 - Texto com muita coerência	5 - Texto com muita coerência	4
1241480232	09/11/201	Feminino	29	Carapicuíb	São Paulo	Sudeste	Ensino Superior	3	1- Muito complexo.	1- Muito complexo.	3	4	5 - Texto com muita coerência	1 - Texto sem coerência nenhuma	5 - Texto com muita coerência	5 - Texto com muita coerência	4
1242614095	12/11/201	Feminino	42	Piraquara	Paraná	Sul	Não Ensino Superior	4	3	4	4	4	4	4	4	4	3
1243183297	10/11/201	Feminino	22	João Pesso	Paraíba	Nordeste	Ensino Superior	4	1- Muito complexo.	2	3	4	5 - Texto com muita coerência	2	3	4	5 - Texto com muita coerência
1243936501	09/11/201	Masculino	28	Maracanaú	Ceará	Nordeste	Ensino Superior	3	1- Muito complexo.	1- Muito complexo.	3	4	5 - Texto com muita coerência	1 - Texto sem coerência nenhuma	4	4	3
1244312717	10/11/201	Feminino	20	São Paulo	São Paulo	Sudeste	Ensino Superior	5 - Muito simples.	1- Muito complexo.	1- Muito complexo.	1- Muito complexo.	5 - Muito simples.	5 - Texto com muita coerência	2	3	2	4
1244382583	09/11/201	Masculino	20	Dourados	Mato Gros	Centro Oes	Ensino Superior	2	1- Muito complexo.	1- Muito complexo.	1- Muito complexo.	5 - Muito simples.	2	1 - Texto sem coerência nenhuma	4	4	3
1244712851	12/11/201	Masculino	47	São Gonçal	Rio de Jane	Sudeste	Não Ensino Superior	1- Muito complexo.	1- Muito complexo.	1- Muito complexo.	1- Muito complexo.	1- Muito complexo.	1 - Texto sem coerência nenhuma	1 - Texto sem coerência nenhuma	1 - Texto sem coerência nenhuma	1 - Texto sem coerência nenhuma	1 - Texto sem coerência nenhuma
1244838580	09/11/201	Feminino	23	Rio de Jane	Rio de Jane	Sudeste	Ensino Superior	5 - Muito simples.	2	3	4	1- Muito complexo.	3	2	4	3	5 - Texto com muita coerência
1247455477	09/11/201	Masculino	18	São Paulo	São Paulo	Sudeste	Ensino Superior	5 - Muito simples.	3	2	4	5 - Muito simples.	5 - Texto com muita coerência	3	5 - Texto com muita coerência	3	1 - Texto sem coerência nenhuma
1248079825	09/11/201	Masculino	37	São Paulo	São Paulo	Sudeste	Não Ensino Superior	4	1- Muito complexo.	3	3	3	3	2	3	3	4
1252232532	09/11/201	Masculino	22	Recife	Pernambu	Nordeste	Ensino Superior	4	1- Muito complexo.	2	3	5 - Muito simples.	4	1 - Texto sem coerência nenhuma	3	4	5 - Texto com muita coerência
1253827887	09/11/201	Masculino	33	Niterói	Rio de Jane	Sudeste	Não Ensino Superior	5 - Muito simples.	1- Muito complexo.	1- Muito complexo.	2	5 - Muito simples.	5 - Texto com muita coerência	1 - Texto sem coerência nenhuma	4	4	5 - Texto com muita coerência
1255400185	10/11/201	Feminino	35	Campinas	São Paulo	Sudeste	Ensino Superior	4	1- Muito complexo.	2	3	4	4	3	3	5 - Texto com muita coerência	5 - Texto com muita coerência
1257151906	10/11/201	Feminino	47	Rio de Jane	Rio de Jane	Sudeste	Não Ensino Superior	2	3	1- Muito complexo.	1- Muito complexo.	2	3	3	3	3	3
1260075552	09/11/201	Masculino	48	Claro dos F	Minas Gera	Sudeste	Não Ensino Superior	5 - Muito simples.	3	1- Muito complexo.	5 - Muito simples.	4	4	5 - Texto com muita coerência	4	2	3
1260963078	10/11/201	Masculino	42	São Leopol	Rio Grande	Sul	Não Ensino Superior	1- Muito complexo.	3	4	2	2	3	5 - Texto com muita coerência	3	3	2
1261382334	09/11/201	Masculino	40	Recife	Pernambu	Nordeste	Ensino Superior	5 - Muito simples.	3	5 - Muito simples.	3	5 - Muito simples.	3	4	5 - Texto com muita coerência	4	4
1261920797	12/11/201	Masculino	22	Campo Ma	Paraná	Sul	Ensino Superior	5 - Muito simples.	1- Muito complexo.	1- Muito complexo.	3	4	5 - Texto com muita coerência	5 - Texto com muita coerência	5 - Texto com muita coerência	5 - Texto com muita coerência	5 - Texto com muita coerência
1263350130	09/11/201	Masculino	37	Ponta Gros	Paraná	Sul	Ensino Superior	4	2	3	3	5 - Muito simples.	4	2	4	3	4
1264063621	12/11/201	Feminino	59	Rio de Jane	Rio de Jane	Sudeste	Não Ensino Superior	4	3	2	1- Muito complexo.	4	4	2	5 - Texto com muita coerência	4	4
1267198780	09/11/201	Feminino	29	Ourinhos	São Paulo	Sudeste	Ensino Superior	3	2	3	3	3	3	4	3	3	3
1268256390	09/11/201	Masculino	26	São Paulo	São Paulo	Sudeste	Ensino Superior	1- Muito complexo.	1- Muito complexo.	1- Muito complexo.	1- Muito complexo.	3	1 - Texto sem coerência nenhuma	3	1 - Texto sem coerência nenhuma	5 - Texto com muita coerência	5 - Texto com muita coerência
